# Corrosion and wear damage estimations for retrieved knee and hip implants

**DOI:** 10.1038/s41529-026-00821-9

**Published:** 2026-06-03

**Authors:** Saman Nikpour, Anastasia M. Codirenzi, Christine Hoskin, Ubong Eduok, Brent Lanting, Matthew G. Teeter, Camila P. E. de Souza, Yolanda S. Hedberg

**Affiliations:** 1 Department of Chemistry, The University of Western Ontario, London, ON Canada; 2 Department of Orthopaedic Surgery, London Health Sciences Centre, University Hospital, London, ON Canada; 3 Department of Medical Biophysics, Schulich School of Medicine and Dentistry, The University of Western Ontario, London, ON Canada; 4 Department of Surgery, Schulich School of Medicine and Dentistry, The University of Western Ontario, London, ON Canada; 5 Department of Statistical and Actuarial Sciences, The University of Western Ontario, London, ON Canada; 6 Surface Science Western, The University of Western Ontario, London, ON Canada

**Keywords:** Diseases, Engineering, Health care, Materials science, Medical research

## Abstract

Predicting the lifetime and function of orthopedic implants requires learning from failures observed in retrieved implants. This study scored mechanical and corrosion damage based on optical microscopy (OM) for 135 trunnion parts of retrieved hip implants and 106 tibial baseplates of retrieved knee implants. We used scanning electron microscopy (SEM) and energy dispersive X-ray spectroscopy to categorize the damage types. This data was correlated with implant and patient factors. Fretting, third-body wear, tribocorrosion, and biofilms were observed. Mechanically assisted corrosion, *e.g.*, tribocorrosion, was the dominant damage mode for both hip and knee implants. The knee implant damage strongly depended on the locking mechanism of the polyethylene insert on the tibial baseplate. For the trunnion part, infection prior to or at surgery was correlated with higher SEM damage scores. Inflammatory arthritis and cemented hip implants correlated with lower SEM damage scores of the trunnion. Damage scores decreased with patient age, and increased with patient weight, body mass index, and years of implantation.

**Figure:**
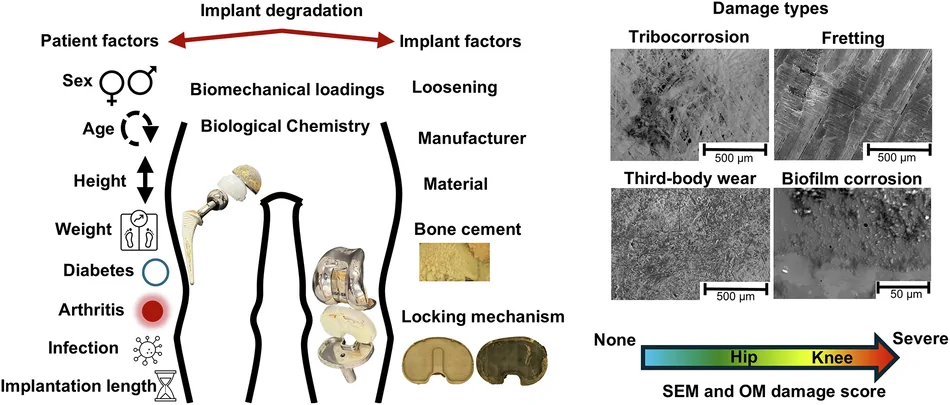

## Introduction

The number of artificial joints is increasing due to, in part, an aging and heavier population and a high frequency of arthritis^1,2^. The main components of hip and knee orthopedic implants are durable metals, polymers, and ceramics, with stainless steel, titanium, and cobalt-chromium alloys being the most prevalent metals used^3,4^. These are so-called passive alloys and form a naturally thin and protective surface oxide layer^5,6^. Despite their high corrosion resistance, they might corrode and release significant amounts of metal ions and particles^7–9^ upon exposure to the harsh chemical and mechanical conditions of the human body. The chemical environment includes aggressive ions (like chloride, phosphate, sulfate, etc.), pH, and proteins^10–13^, while mechanical stresses cause the destruction of the protective surface oxide through micromotions, rubbing, third-body wear, and fretting^14,15^. The degradation and longevity of metallic implants depend on a system (material and environment), which is influenced by a combination of implant properties, such as material composition, microstructure, manufacturing process, and design^16–19^, as well as patient-specific factors, such as age, weight, height, implantation length, infection, and diabetes^20–23^. Material surface reactions can trigger environmental chemical changes, e.g., decreased local pH or a deaerated crevice^24^, which ultimately worsen the corrosion, metal release, and subsequent adverse local tissue reactions^25^. All these phenomena might accelerate the implant failure and the need for revision surgery^26^.

Implant retrieval studies are invaluable for investigating this dynamic interplay between patient and material factors. Retrieval studies highlighted the effect of alloy choice^27^, design parameters like femoral head size or modularity, which affect micromotions^28,29^, and the manufacturer’s approach to implant production^30^. Regarding patient factors, the density and structure of bone are important and depend on age and sex^31^. A higher body weight is linked to greater mechanical loads and more aseptic loosening, which makes it a better predictor for arthroplasty survival than body mass index (BMI)^20^. Conditions like arthritis or diabetes may alter the chemistry of the implant’s surrounding environment^32,33^. A wide range of corrosion and mechanical damage mechanisms, individually or synergistically, have been reported for the retrieved metallic systems^34^. Depending on the geometry, design, application, applied load, and counter bodies, these modes of damage have been identified on different parts, for instance, the modular head-neck taper interface (trunnion part)^30^, femoral head^35^ of hip implants, or the femoral condyle^36^, tibial baseplate and sleeve^37,38^ of knee implants. Polymeric inserts can also degrade and influence the degradation of the metallic parts. For knee implants, the continuous rubbing between the femoral condyle or tibial baseplate and polyethylene inserts, which deteriorates the polymeric inserts and produces particles and/or relatively worn-through inserts, is a condition known as backside wear^39^.

Goldberg et al.^40^ proposed a scoring system for the visual quantification of the corrosion and wear severity of the trunnion part of hip implants. A similar scoring system has been adapted for the damage scoring of different knee components^27,38^. The tibial baseplate experiences continuous and cyclic mechanical forces^37^. However, most damage scoring approaches rely on optical imaging and do not identify the underlying damage mechanism.

This study (1) compared Goldberg scores of hip trunnion parts with adapted Goldberg scores for knee tibial parts, (2) compared scores from optical microscopy (OM) and detailed SEM and energy dispersive X-ray spectroscopy (EDS) analysis of damage types, and (3) correlated these damage scores with implant factors (manufacturer, implant system, material, design, loosening, instability, bone cement) and patient factors (sex, age, weight, height, implantation length in years, BMI, infection, pain, inflammatory arthritis, diabetes) for a total of 241 hip and knee implants retrieved in Canada.

## Results

### Damage scores based on optical microscopy

**Fig. 1:**
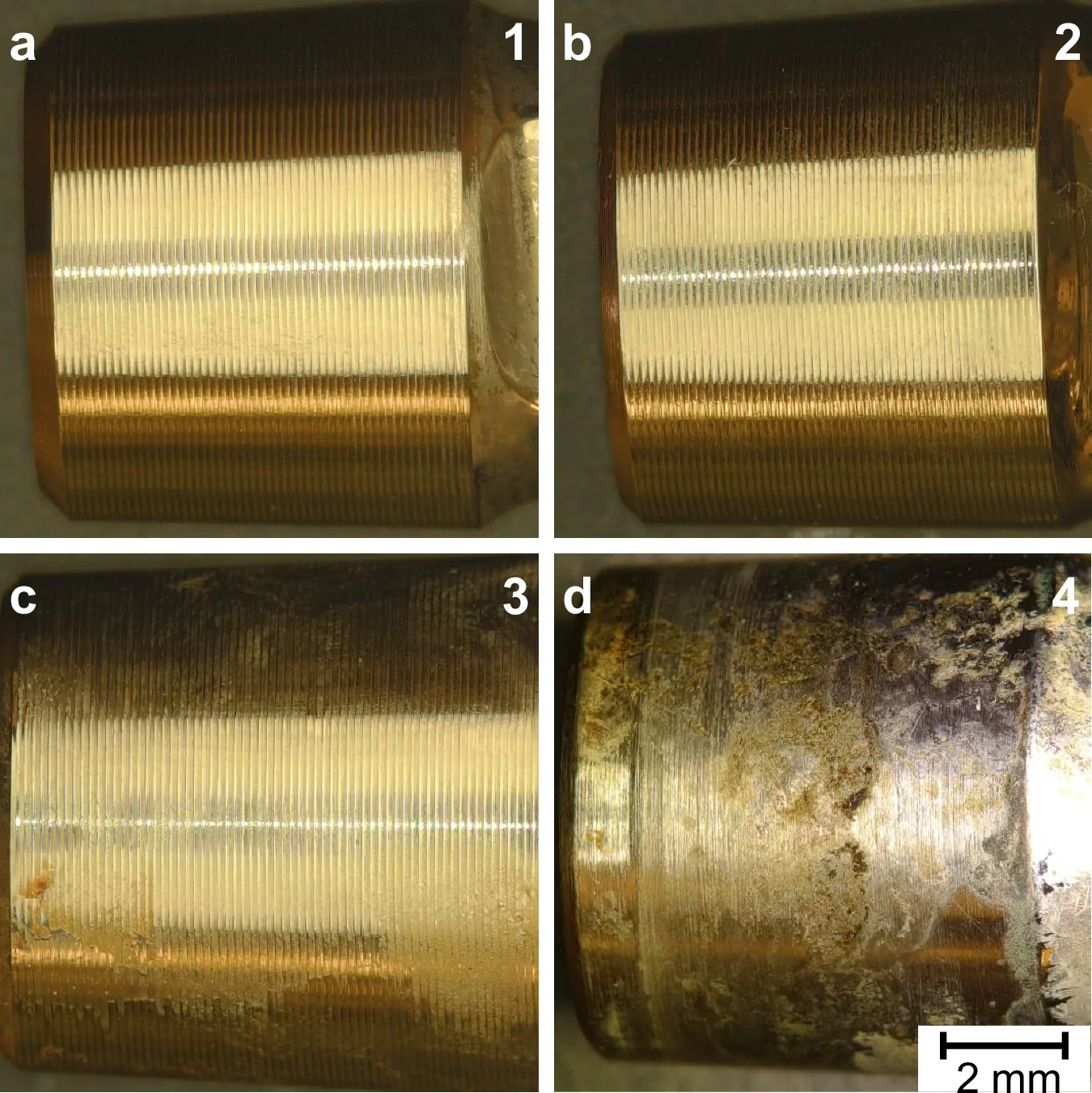
The OM analysis of hip trunnion parts. Examples of each damage score: **a** none (score 1), **b** mild (score 2), **c** moderate (score 3), and **d** severe (score 4), for the trunnion part of the hip implant according to the Goldberg method (Table 1). The scores are written in the top right of each figure.

**Fig. 2:**
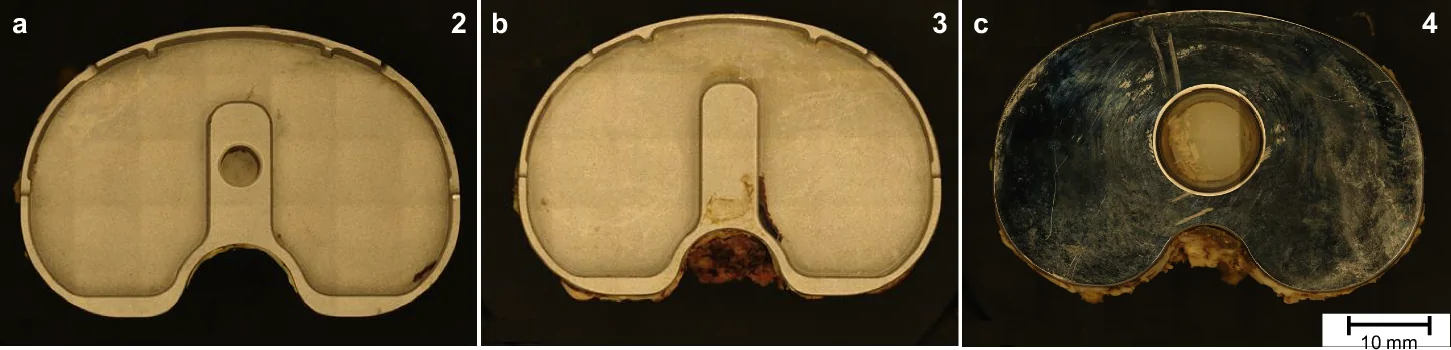
The OM analysis of the tibial baseplates. Examples of each damage score: **a** mild (score 2), **b** moderate (score 3), and **c** severe (score 4), for the tibial baseplate of the knee implant according to Table 1. The scores are written in the top right of each figure.

Figures 1 and 2 depict examples of OM images representative of each score for hip and knee implants, respectively. Each number in Fig. 1 shows the corresponding damage score (top right) according to Goldberg’s criteria (Table 1). Figure 1a, as a representative of score 1, shows almost no signs of corrosion or wear. There was no significant discoloration or wear lines on the surface. A slight change of color and a few wear scars were counted as mild damage on the surface for score 2, Fig. 1b. For score 3, more discoloration and worn-away signs were seen, Fig. 1c, which describes a moderate degradation. Figure 1d (score 4) displays severe corrosion attacks and probably wear scars, which might have been smeared out by corrosion products adhered to the surface. The yellowish color of the implants is related to the light settings of the OM.

**Table 1:** Damage score criteria for the tibial component of the knee implant and femoral stem trunnion of the hip implant

Damage intensity	Score	Tibial component	Trunnion^40^
None	1	No visible corrosion observed. No visible scratches observed.	No visible corrosion observed. No visible signs of fretting observed.
Mild	2	<25% of the surface discolored or dull. Single or several scratches in at least one quadrant.	<30% of taper surface discolored or dull. Single band or bands of fretting scars involving three or fewer machine lines on the taper surface.
Moderate	3	>25% of the surface is discolored or dull, or <25% of the surface contains black debris, pits, or etch marks. Several scratches in at least two quadrants.	>30% of taper surface discolored or dull, or < 10% of taper surface containing black debris, pits, or etch marks. Several bands of fretting scars or a single band involving more than three machine lines
Severe	4	>25% of the surface contains black debris, pits, or etch marks. Several scratches on the whole surface.	> 10% of taper surface containing black debris, pits, or etch marks. Several bands of fretting scars involving several adjacent machine lines, or flattened areas with nearby fretting scars

Figure 2 exhibits different damage scores, from 2 to 4, for the tibial component. These scores were assigned based on the criteria in Table 1. Since signs of degradation and surface damage were evident on the surface of all studied tibial parts, there was no intact and undamaged surface, so no implant was assigned a score of 1. Figure 2a shows discoloration and scratches in the top left quadrant (mild, score 2). In Fig. 2b, the presence of scratches and discoloration was on more than two quadrants of the surface, implying moderate damage severity (score 3). Accordingly, a heavily damaged surface could be seen in Fig. 2c, accompanied by extensive debris and scratches (severe, score 4). The blackish color of the tibial baseplate in Fig. 2c is related to the light reflection from the polished surface.

### Damage modes and severity identified by scanning electron microscopy

**Fig. 3:**
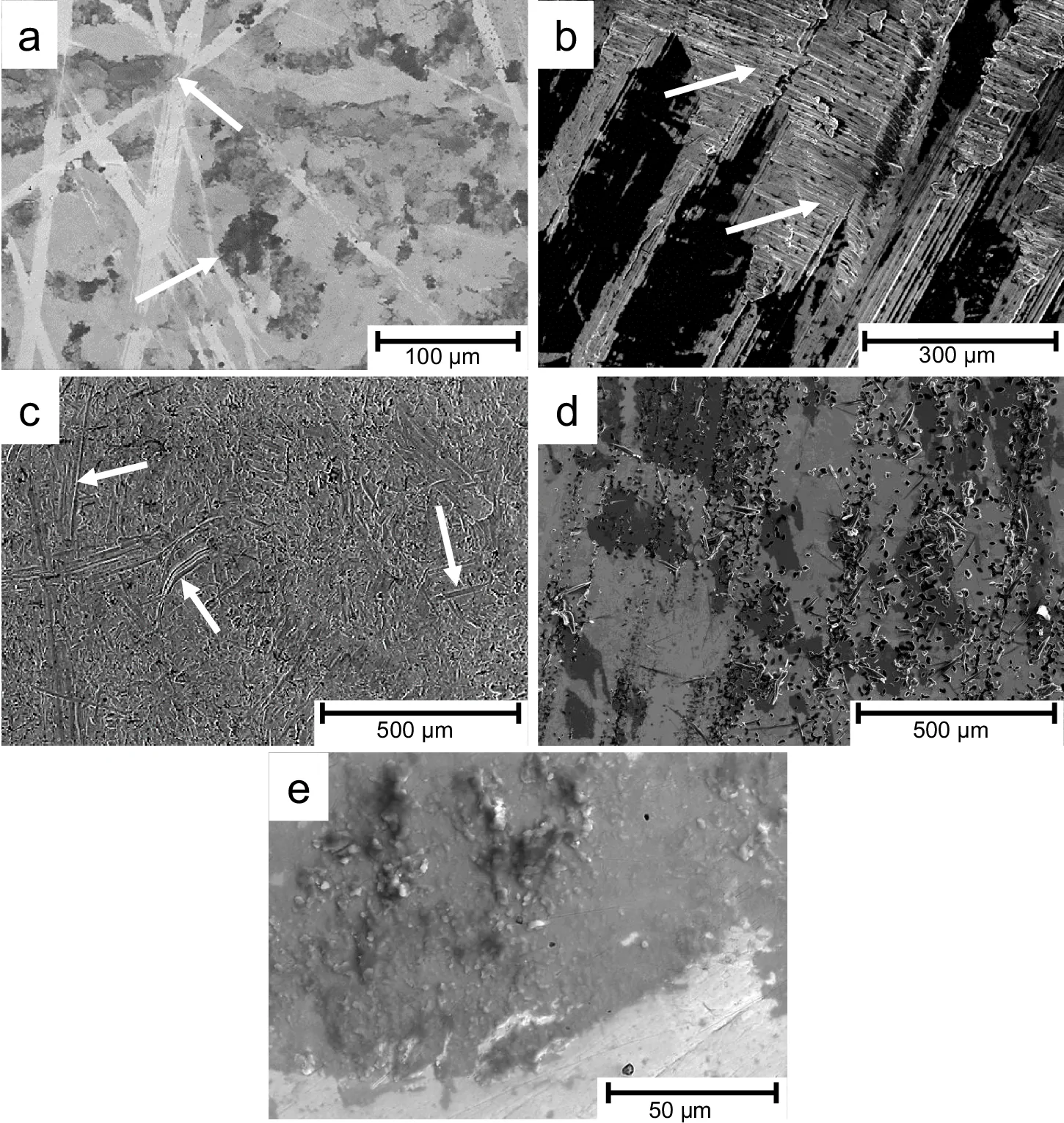
Different damage modes observed by SEM. **a** Example of tribocorrosion of the tibial part of a knee implant (CoCrMo alloy); the tribochemical layers are shown by white arrows, **b** example of fretting of the trunnion region of a hip implant (Ti6Al4V alloy); white arrows show fretting lines, **c** example of third-body wear on the surface of the tibial component of a knee implant (Ti6Al4V alloy); white arrows show deep scratches generated by third bodies, **d** example of adsorbed/adhered organic species on the surface of the tibial component of a knee implant (CoCrMo alloy), and **e** example of biofilm-induced corrosion on the surface of the tibial component of a knee implant (CoCrMo alloy).

SEM and EDS were used to assign the damage modes for the SEM scores, with examples including EDS analyses shown in Figs. S3–S7. An example of mechanically assisted corrosion (tribocorrosion) for the tibial baseplate is shown in Fig. 3a. Wear signs were elongated across the surface. Figure S3 demonstrates the enrichment of chromium and molybdenum in the depleted cobalt spots, indicating the occurrence of corrosion^41^. Based on the elemental distribution (Fig. S3), carbon and oxygen were co-located, which is possibly related to the attachment of organic species mixed with corrosion products (carbon-rich tribolayer^42^).

Mechanical motion at the microscale, capable of destroying the passive film, is called fretting or fretting corrosion (with a synergistic effect with corrosion)^43^. An example of small-scale scars (white arrow) from fretting is exhibited in Fig. 3b for the trunnion part. Compared to tribocorrosion, wear tracks were smaller. This micromotion occurred between the trunnion and femoral head, leading to surface oxide disruption, followed by corrosion and re-passivation^44^. This type of damage (fretting) was not detected for any knee implants in the sample pool of this study. Knee implants were subjected to harsher conditions and typically underwent tribocorrosion or large-scale motions.

Third-body wear occurs when wear debris (particles) becomes trapped between two contacting surfaces, causing mechanical damage and potentially leading to corrosion of the exposed surface. Third-body wear was observed in both knee and hip implants. Figure 3c illustrates this mode of damage for the tibial baseplate. Hard particles, such as metallic debris and bone cement, can become lodged between contacting surfaces and induce abrasive wear. Due to the geometry of the knee implant, this mode of damage was more common for the tibial part. Deep scratches in arbitrary directions were present on the surface, which implied the third-body particle’s movement between the two surfaces.

Organic species originating from cells, biofilms, or protein layers were typically present on most of the implants. An example of adhered organic species on the surface is shown in Fig. 3d. Corresponding EDS maps (Fig. S6) show the simultaneous presence of C, O, and Na, indicative of organic species. Concomitant Na, Ca, and P were attributed to the presence of bone cells. Other organic deposits contained only C and O, or C, N, and O. The organic deposits’ morphology differed considerably, reflecting different cell types. Although a large fraction of both hip and knee implants showed organic deposits, there were no signs of localized corrosion, such as enrichment or depletion of any elements in the presence of the organic species. Only in one case, for one knee implant shown in Figs. 3e and S7, chromium and molybdenum were locally enriched beneath the biofilm. The enrichment of chromium and molybdenum was previously shown as a result of the Fenton reaction (including iron and hydrogen peroxide species), but in our case, no iron was found^45^. This indicates that this corrosion case was caused by a different cell- or biofilm-induced mechanism, such as other extracellular substances, a local pH decrease, or a crevice beneath the biofilm.

**Fig. 4:**
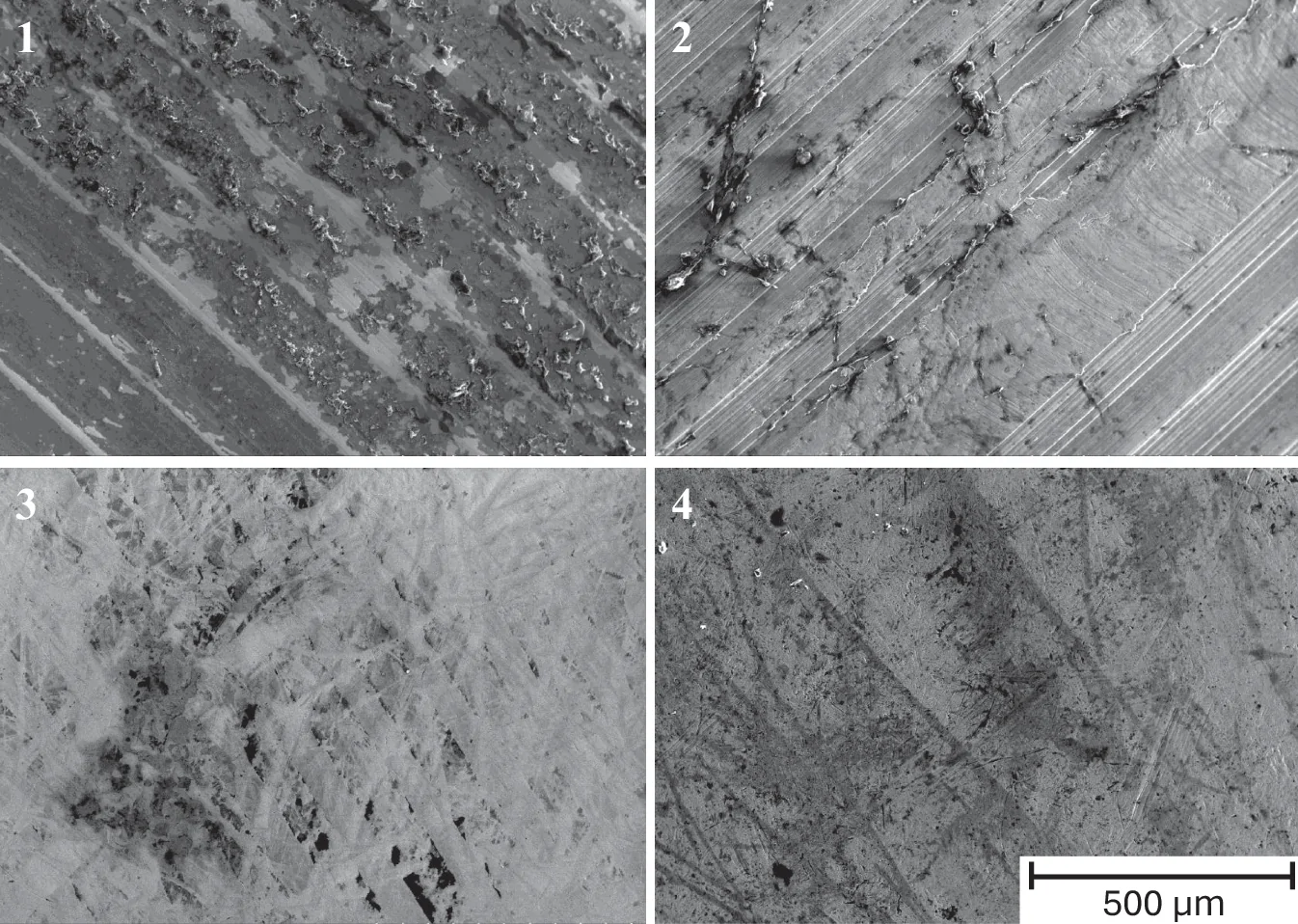
Damage scores based on SEM images. Examples of each SEM damage score: (1) none, (2) mild, (3) moderate, and (4) severe, according to Table 2. The scores are written in the top left of each figure.

Figure S8 displays the prevalence of the above-introduced damage modes for knee and hip implants imaged by SEM. We also assigned SEM scores based on the criteria in Table 2. The top-left corner of Fig. 4 shows examples of various SEM scores. Score 1 (Fig. 4.1) displays organic deposition without specific signs of corrosion or mechanical damage (none). Score 2 means that at least one type of corrosion or mechanical damage occurred on the surface, exemplified in Fig. 4.2 as fretting (mild). Score 3 means that two types of damage (corrosion and mechanical) occurred on the surface (moderate), exemplified in Fig. 4.3, which shows both large-scale motions and corrosion (tribocorrosion). The most severe damage score (score 4) was the combination of tribocorrosion and third-body wear, as illustrated in Fig. 4.4. In this case, several types of damage were detected on the surface.

**Table 2:** Damage score criteria for SEM images of both hip and knee implants

Damage intensity	Score	Characteristics
None	1	Organic deposition without signs of corrosion or physical damage
Mild	2	At least one type of corrosion or physical damage
Moderate	3	Presence of corrosion and physical damage (mechanically assisted corrosion, *e.g.*, tribocorrosion or fretting corrosion)
Severe	4	More than one type of corrosion and physical damage

### Correlations of implant and patient factors with corrosion and damage scores

The SEM or OM damage scores of knee implants were correlated with all the investigated implant and patient factors (Table S1). Out of the 15 patient factors and their binned factors, only “infection 6 weeks after surgery” was statistically correlated with the SEM damage score of the knees. Of the 8 implant factors, only “implant manufacturer” and “implant system” were significantly correlated with the OM damage score in the knees. These significant correlations are discussed below. Since all implant systems have their own manufacturer, only the implant systems are included in the analysis below.

**Fig. 5:**
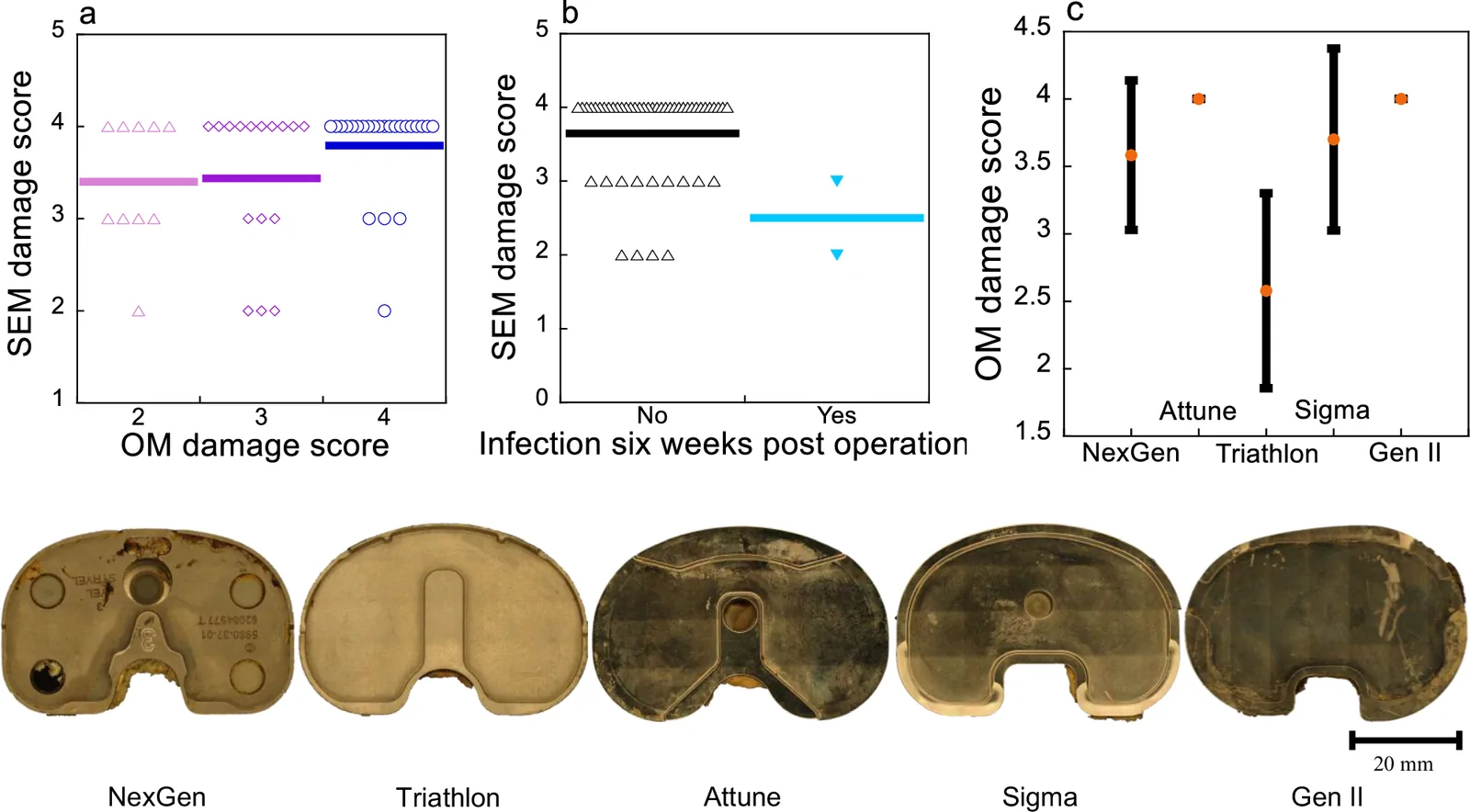
Significant correlations identified for the tibial baseplate. **a** Correlation between SEM and OM damage scores of knee implants, **b** correlation between SEM damage scores of knee implants and infection 6 weeks post-operation, and **c** correlation between OM damage scores and implant system. Gen II: Genesis II.

There was also a strong link between OM and SEM damage scores (*ρ* = 0.289, *n* = 50, *P* = 0.042), suggesting that a tibial baseplate with a higher OM score also likely had a higher SEM corrosion/damage score (Fig. 5a). Figure 5b shows that two patients who experienced an infection after 6 weeks post-operation had tibial baseplates with less SEM score damage severity (*U* = 10.50, *Z* = −2.30, *P* = 0.021) than those who didn’t have a post-operative infection; however, since this is only based on two patients, this is not further discussed or considered. An insignificant positive association (longer service life means higher SEM damage score) was observed between SEM damage scores and implantation length (Fig. S9a, *T* = 3.057, df = 1, *P* = 0.080). Figure S9b shows that there was also a non-significant trend that patients with post-operative infection after 6 weeks experienced a shorter implantation length (*U* = 109, *Z* = −0.667, *P* = 0.505).

The OM damage scores of tibial baseplates appeared to depend significantly on various implant systems, as shown in Fig. 5c. Implant systems are different among various manufacturers (Fig. S10). The Kruskal–Wallis test analyzed 106 knee implants and revealed significant differences among systems (*H* = 50.36, df = 4, *P* < 0.001). Results showed that Triathlon had the lowest damage scores among implant systems. However, the number of each system varied: 36 NexGen, 11 Attune, 45 Triathlon, 10 Sigma, and 4 Genesis II.

Among the 15 patient factors and their binned factors, three significantly correlated with SEM damage scores of hip implants: weight (binned), arthritis, and infection prior to or at surgery (Table S2). Among the 9 implant factors, only “hip stem cemented” showed a significant correlation with the SEM damage scores of the hip implants. These are discussed below.

**Fig. 6:**
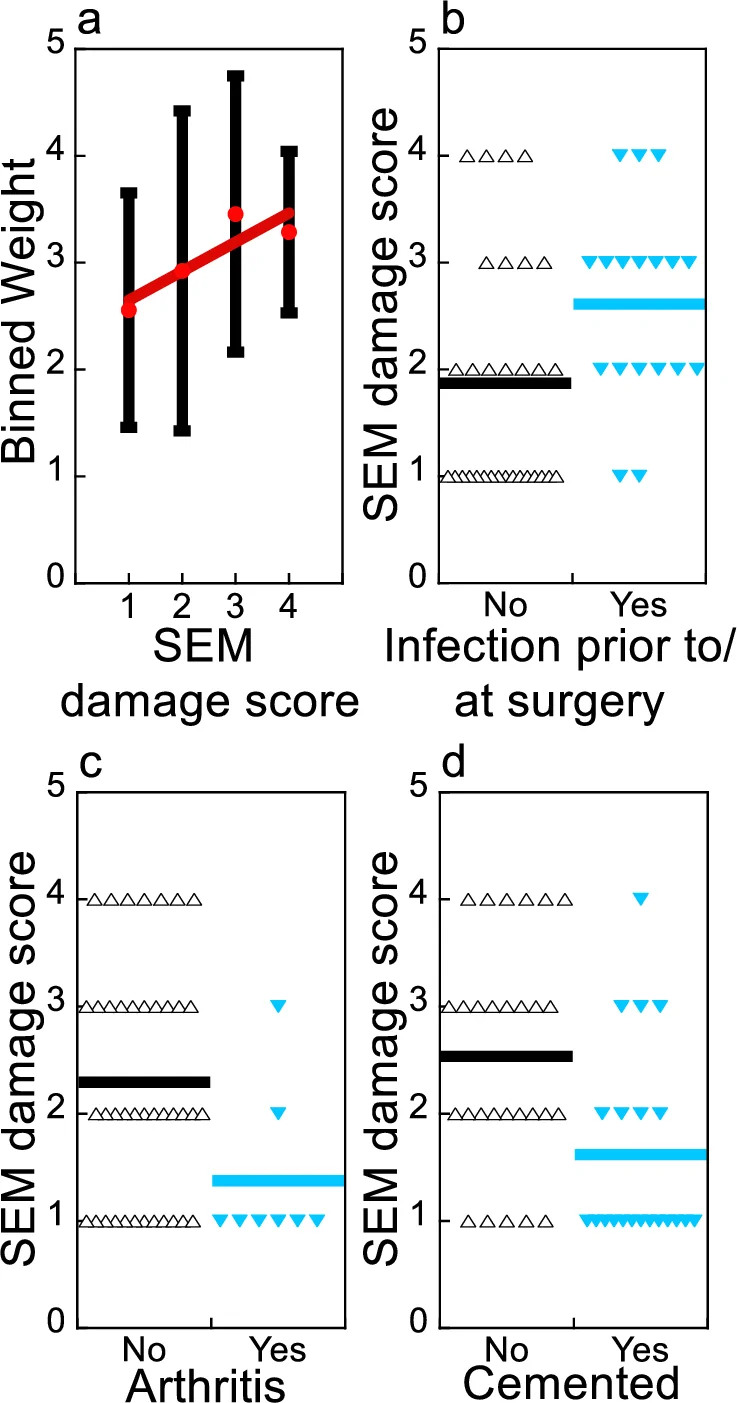
Significant correlations identified for the hip trunnion. **a** Correlation between SEM damage scores of hip implants (trunnion) and binned weight (*y* = 2.37 + 0.27*x*, *R* = 0.88, regression equation based on mean values), **b** correlation between SEM damage scores of hip implants and infection prior to/at surgery, **c** correlation between SEM damage scores of hip implants and inflammatory arthritis, and **d** correlation between SEM damage scores of hip implants and bone cement status.

Significant correlations between damage scores and various factors for the trunnion part of hip implants are presented below. Figure 6a shows that the SEM damage scores of the trunnion part were significantly positively correlated with binned weight, meaning that a heavier weight resulted in more corrosion and mechanical damage (*ρ* = 0.331, *n* = 49, *P* = 0.020), due to more mechanical stresses^20^. Figure 6b depicts that an infection prior to or at surgery resulted in more corrosion or mechanical damage (*U* = 163, *Z* = −2.51, *P* = 0.012). According to Figure S11a, there appeared to be a trend of longer implantation length with a smaller SEM damage score, but this was not significant (*T* = 2.003, df = 1, *P* = 0.157). Figure S11b shows that patients with infection at/prior to surgery experienced a shorter implantation length (*U* = 1173.5, *Z* = −2.13, *P* = 0.033). Figure 6c shows that hip implants in patients with inflammatory arthritis had lower SEM damage scores than those without inflammatory arthritis (*U* = 83.0, *Z* = −2.28, *P* = 0.022). The bone cement status (yes or no) was the only studied implant parameter that led to significant differences in the SEM damage scores for the trunnion part of hip implants. Figure 6d shows that the application of bone cement resulted in less corrosion damage, which was significantly correlated (*U* = 149.5, *Z* = −3.047, *P* = 0.002). Since having an infection prior to/at surgery and inflammatory arthritis were the patient factors impacting the corrosion of hip implants most significantly, their associations with cement status were also studied and are depicted in Fig. S12a, b, respectively. Implants with bone cement had a lower percentage of infection prior to/at surgery (*χ*^2^ = 5.085, df = 1, *P* = 0.024) and a lower, but not statistically significant, percentage of inflammatory arthritis conditions compared to cementless implants (*χ*^2^ = 0.258, df = 1, *P* = 0.612).

Patient factors like age, sex, BMI, and diabetes did not show any significant correlations with individual damage scores of either knee or hip implants (Tables S1 and S2), which might be due to too low a number of implants investigated in each group. When the hip and knee implants were pooled together, some correlations with patient factors became statistically significant: Patient age, weight, BMI, and implantation length (years of service of the implant). As these patient factors are expected to influence both hip and knee implants of a patient, they are discussed below.

**Fig. 7:**
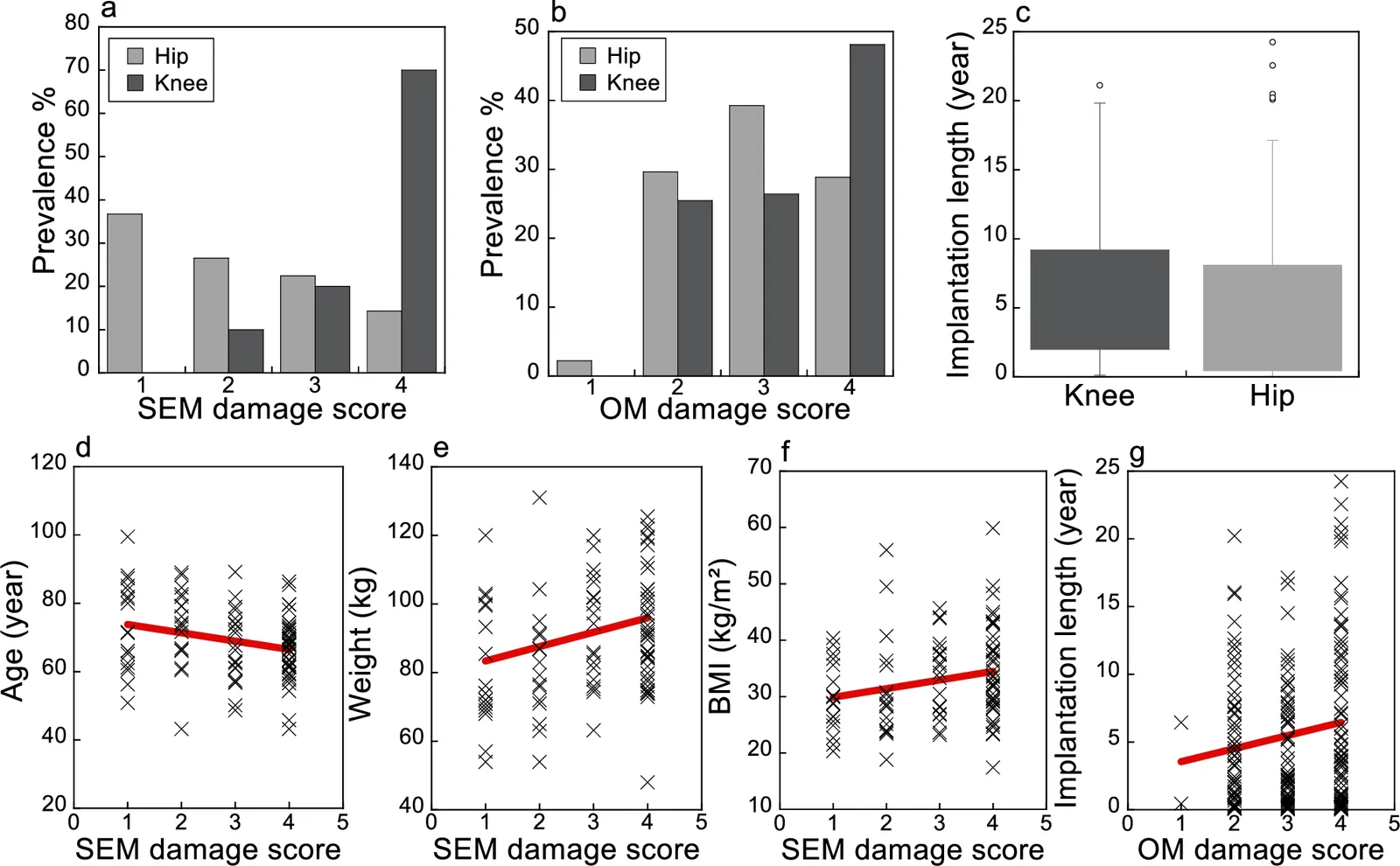
The prevalence of damage scores for hip and knee implants and significant correlations with patient factors. **a** SEM damage scores and type of implants, **b** OM damage scores and type of implants, **c** implantation length in years and type of implants, **d** correlation between SEM and age (*y* = 76.20−2.43*x*, *R* = 0.26, regression equation), **e** correlation between SEM and weight (*y* = 79.188 + 4.19*x*, *R* = 0.22, regression equation), **f** correlation between SEM and BMI (*y* = 28.34 + 1.53*x*, *R* = 0.22, regression equation), and **g** correlation between OM and implantation length (*y* = 2.58 + 0.96*x*, *R* = 0.14, regression equation).

The knee and hip implants are not directly comparable, and the studied corroded/worn parts of them (trunnion and tibial baseplate) are exposed to different mechanical and chemical environments. This was reflected in the fact that the SEM damage scores were significantly different between knee and hip implants (*U* = 370, *Z* = −6.30, *P* < 0.001) and were higher for knee implants than hip implants (Fig. 7a) in a way that no knee implants received a score of 1, while the SEM scores of hip implants ranged from 1 to 4. Likewise, the corrosion severity based on OM images was significantly different between implant types (*U* = 5852, *Z* = −2.57, *P* = 0.01) and was higher for knee implants (Fig. 7b) than for hip implants. Given the higher damage scores for knee than hip implants, we investigated whether this was true for all locking mechanisms or only certain ones. The results (Tables S3–S7, Fig. S13) are presented in detail in the SI and show that only the optimized design of the Triathlon system could, on a macroscopic level, limit wear and corrosion compared with less-loaded hip implants.

Implantation length (years of implantation) is one of the important factors to consider for damage severity. There was a significant difference in implantation length between knee and hip implants (*U* = 4175, *Z* = −2.89, *P* = 0.004), which reflects a longer service life of knee implants, resulting in higher damage scores (Fig. 7c). The implantation length of each knee implant system was compared with the implantation length of hip implants in Fig. S14. Except for the Attune system, all other knee implant systems had a longer service life inside the body than hip implants.

SEM damage scores (across both hip and knee implant types) were significantly associated with age, weight, and BMI, while OM damage scores were significantly associated with implantation length. The association between SEM damage scores and age (Fig. 7d) shows an inverse trend, indicating that younger patients have greater surface damage severity (*T* = 6.488, df = 1, *P* = 0.011). Conversely, there was a positive trend between SEM damage score and weight shown in Fig. 7e (*T* = 3.988, df = 1, *P* = 0.046). Similarly, there was a significant association between BMI and SEM damage scores (Fig. 7f), indicating that higher BMI was associated with greater SEM damage (*T* = 4.630, df = 1, *P* = 0.031). Additionally, there was a positive association between OM damage scores and implantation length (*T* = 5.280, df = 1, *P* = 0.022), suggesting that the longer an implant is inside the body, the higher the expected damage score (Fig. 7g).

## Discussion

Our finding of an inverse correlation of damage scores of both hip and knee implants with age can be explained by the fact that younger people’s greater activity may increase the frequency of mechanical cycles, leading to more mechanically assisted corrosion. This study also showed an overall role of weight on damage scores of both hip and knee implants. Higher weight and BMI of patients were reported to increase the incidence of corrosion of implants^35,46^. We also found an overall effect of the service life (implantation length) for the pool of hip and knee implants. This is in agreement with earlier implant retrieval studies^28,40,47^. We did not find any other global correlations with investigated patient factors, including sex, diabetes, height, number of revisions, diabetes, implant loosening, implant instability, and pain. A higher incidence of aseptic loosening and a lower survival rate for hip and knee implants were previously found to be correlated with a younger age and higher weight^20^, which is consistent with our findings. However, infection and arthritis were correlated with the damage scores of hip implants in this study, as discussed further below.

Tribocorrosion, third-body wear, and fretting (only for hip implants) were the most common damage modes in this study. The design factors and biomechanical conditions that affect wear differ between the here-studied parts (trunnion and tibial baseplate) of hip and knee implants, which are therefore discussed separately, or in context, below.

The tibial baseplate of the knee implant wears between a metal and a polymer surface, where locking mechanisms govern backside wear and associated surface damage, often related to third-body wear particles (Fig. S8). According to the OM and SEM analyses of the tibial baseplate, the extent of damage scores was directly dependent on the locking mechanism of the tibial baseplates. For instance, score 4 (Fig. 2c) was associated with a freely rotating system without fixation, which most likely increased backside wear, leading to intense motion between the polyethylene and tibial surfaces, potentially triggering instability^48^. This freedom of motion can facilitate third-body action by other trapped particles, like bone cement or metallic wear particles, between the tibial baseplate and the polyethylene surface. On the other hand, the example of score 2 (Fig. 2a) represents a full peripheral-rim capture with a central anti-rotational island locking mechanism that provided a strong fixation between the tibial baseplate and the polymeric insert, causing reduced wear and corrosion rates^49,50^.

An optimized locking mechanism between the tibial baseplate and the polymeric inserts appeared to effectively hinder large-scale motions. Based on the various systems depicted in Figs. 5 and S10, the partial peripheral-rim capture design of Attune (also a freely rotating system) and Genesis II resulted in increased damage, likely due to the enhanced motion of the insert and, consequently, external trapped particles. Attune (*n* = 9) was the knee implant system that had the shortest service life (3.5 years of implantation), even shorter than the here-studied hip implants, indicating that its high damage score is associated with clinical consequences. The presence of a central anti-rotational island in the Triathlon system enhanced fixation, resulting in less damage than in the Sigma system, while both systems achieved full peripheral-rim capture. Although the NexGen system achieved greater fixation by using screws, it suffered more corrosion damage due to preferential sites for localized corrosion and the accumulation of wear particles. The backside wear was initially one of the main failure reasons; however, recent advancements in the manufacturing of polyethylene insert (highly cross-linked), implant design (locking mechanism), and simulative studies have significantly overcome this issue^51–53^.

Both SEM and OM damage scores showed greater damage intensity in the knee than in the hip implants. When the effect of implantation length was controlled for, the SEM damage scores remained significantly associated with implant type (ordinal regression analysis, *P* < 0.001), suggesting that the tibial part of knee implants experienced overall harsher conditions than the trunnion and exhibited more severe surface damage. However, this was not true for OM scores, which were not significantly associated with implant type (hip or knee) after controlling for implantation length (ordinal regression analysis, *P* = 0.097). Tables S3–S7 and Fig. S13 showed that SEM/OM damage scores remained significantly higher for knee implants when tibial baseplates with the NexGen and Attune systems were compared individually with those of hip implants, whereas all other systems showed mixed or non-significant differences. This implies that quantitative differences in damage scores between the tibial baseplates and the trunnion part of the hip are explained by the locking mechanism of the baseplate.

This study cannot answer the question of material influence on damage scores of the tibial baseplate, since the majority of studied tibial baseplates were made of CoCrMo (*n* = 83), with only one titanium alloy, and missing data for the remainder, making meaningful statistical analysis impossible. We didn’t find any correlation of damage scores with the femoral and tibial sizes despite relatively good statistical power (information available for *n* = 27 OM and *n* = 16 SEM femoral and *n* = 84 OM and *n* = 47 SEM tibial components). In addition, there was no correlation found between any damage scores and femoral designs, including Antero-Posterior (A/P) and Medio-Lateral (M/L), because this data was missing for most of the implants. Although significant correlations were not identified in this study, previous studies highlighted how different material properties (e.g., stiffness, load transfer, and shape) can impact stress shielding, bone resorption, backside wear, and aseptic loosening in total knee arthroplasty^54–57^.

The hip implant is a neck metal surface against a metal or ceramic surface, where the extent of degradation is strongly dependent on the femoral head features. Having an infection before the total hip joint replacement caused more corrosion/wear damage in a shorter period of implantation. An infection during or prior to revision surgery might have led to a quicker revision or a shorter implantation length due to the presence of bacteria, which then affected the functionality and implantation length of a total hip joint arthroplasty^58,59^. The preexisting infection might have started attacking the hip implant’s surface after implantation, leading to early revision, or, alternatively, the absence of infection might have allowed a longer implantation with less damage than if infection were present. Differences in SEM damage scores between having an infection prior to/at surgery remained statistically significant even after controlling for the effect of implantation length (ordinal regression analysis, *P* = 0.037). The association also remained statistically significant after controlling for other patient factors, except weight (ordinal regression analysis, *P* = 0.057).

The lower SEM damage scores for the hip trunnion under conditions of inflammatory arthritis might be related to the highly oxidative condition (caused by the inflammation), followed by faster repassivation during mechanically assisted corrosion^60^, and/or, more likely, a lower extent of activity for these patients due to chronic pain^61^. However, after controlling for implantation length (patients with arthritis had slightly longer implantation length than those without arthritis), SEM damage scores and inflammatory arthritis were no longer statistically significantly correlated (ordinal regression analysis, *P* = 0.089).

It has previously been shown that the application of bone cement can provide greater stability and enhance the service life of hip implants used in patients with arthritis by reducing the risk of fractures^62^. Also, bone cement can delay the access of body fluids to the implant surface and chemical degradation. After controlling for the effect of implantation length, differences in SEM damage scores between bone cement status groups remained statistically significant (ordinal regression analysis, *P* = 0.045). Also, the correlation remained statistically significant when controlling for other patient factors. Moreover, the use of bone cement in older patients with poor bone quality may reduce fracture risk and extend implant longevity. Usually, older patients have lower activity compared to younger patients, which could explain the lower SEM damage score. However, in our study, the number of cemented hip implants was not significantly higher in older patients than in younger patients (*P* = 0.318).

In this study, no effect of implant factors other than cementation was found on hip implant damage scores. These implant factors include the hip stem offset, collar, stem material (37 cobalt-chromium alloys, 82 titanium alloys, and 6 stainless steel), hip head material (124 cobalt-chromium alloys, 6 ceramic), model, and size. However, it has been reported that both femoral head and stem characteristics, including size, design, and material, can directly influence the degradation of hip implants^63,64^.

Retrieved components usually exhibit signs of concurrent degradation mechanisms (Figs. S3–S8). In this study, OM and SEM damage score analysis compared the extent of corrosion and mechanical damage that regularly occurred on the surface, but this study did not analyze the root-cause failure reason or estimate the damage score’s importance for clinical outcomes. For example, it has previously been reported that taper-related corrosion of the taper junction of hip implants did not correspond to taper failure^65^. However, the finding that one of our most-worn tibial baseplate systems also had a significantly shorter service life underscores the importance of degradation studies.

Overall, due to differences in service conditions and driving degradation factors between hip and knee implants, their damage scores do not reflect their relative performance. For instance, instability can result from a lack of cement fixation in hip implants, while backside wear could be responsible for instability in knee implants. Additionally, instability may cause varying degrees of damage to the tibial baseplate and trunnion surfaces, limiting their comparison. Thus, a higher damage score on the surface of each component is not necessarily indicative of inferior performance, and this study did not investigate clinical outcomes of the full implant systems. The SEM damage score criteria introduced in this study can, however, be further informative for the extent and mechanism of the damage. The sample size for the SEM damage/corrosion scores was only 99, as opposed to 241 for the OM damage/corrosion scores. Also, the sample size for a single implant type (hip or knee) was even smaller (about half), and the corrosion mechanisms were specific to that type, limiting the ability to identify important patient factors. Future studies with large sample sizes and a single implant type will be most valuable for identifying patient factors that influence outcomes.

It should also be emphasized that this study focuses solely on explanted implants, which constitute a small percentage of the total. This study, therefore, cannot provide any indication of overall success and only contributes to improving the worst cases. A true control group would have retrieved (non-failed) implants post-mortem.

The limited variability of critical covariates, such as bearing components, and missing or incomplete clinical, patient, and implant data reduced the power of statistical analysis in this study, thereby disabling some conclusions about potentially clinically and mechanistically important factors. This study was also limited by its sample selection and size. If this study found no significant correlation for certain patient factors, such as diabetes, it might be due to the sample size and selection, and does not mean there is no correlation. Future studies should also correlate any concomitant tissue damage with the extent of corrosion through tissue or blood analysis.

Moreover, this study cannot determine the effect of surgical protocols. Generally, surgical protocols, such as insertion methods, cleaning protocols, and various angles, can influence the degradation at the interfaces between the bone and implant surface, the bone and cement surface, or the cement and implant surface. Insufficient cleaning of taper junctions may assist the entrapment of biological materials and create a condition for enhanced susceptibility to fretting and corrosion.

In summary, this study analyzed the extent of corrosion and wear damage of the trunnions of hip implants and the tibial baseplates of knee implants using OM and SEM and correlated these scores with implant and patient factors. The following main conclusions were drawn:

1. Fretting was only found on the trunnion part of the hip implant. Tribocorrosion was the predominant type of damage for both implants. Third-body wear was primarily observed on the tibial baseplate of the knee implant.
2. The damage to the knee implant strongly depended on the locking mechanism of the polyethylene insert on the tibial baseplate. The optimized locking mechanism of the Triathlon knee implants was the only knee implant type that showed lower damage scores (by OM) than hip implants.
3. The extent of corrosion/wear damage for both hip and knee implants decreased with patient age, and increased with patient weight, BMI, and years of implantation.
4. For the trunnion part in the hip implant, infection prior to or at surgery was correlated with higher SEM damage scores, even if corrected for potential confounders. Inflammatory arthritis and cemented hip implants correlated with lower SEM damage scores. Patients with cemented hip implants also had a lower infection rate at surgery.
5. SEM damage scores were more distinctive than OM damage scores in the correlation with patient and implant factors.

## Methods

### Retrieved implants

**Fig. 8:**
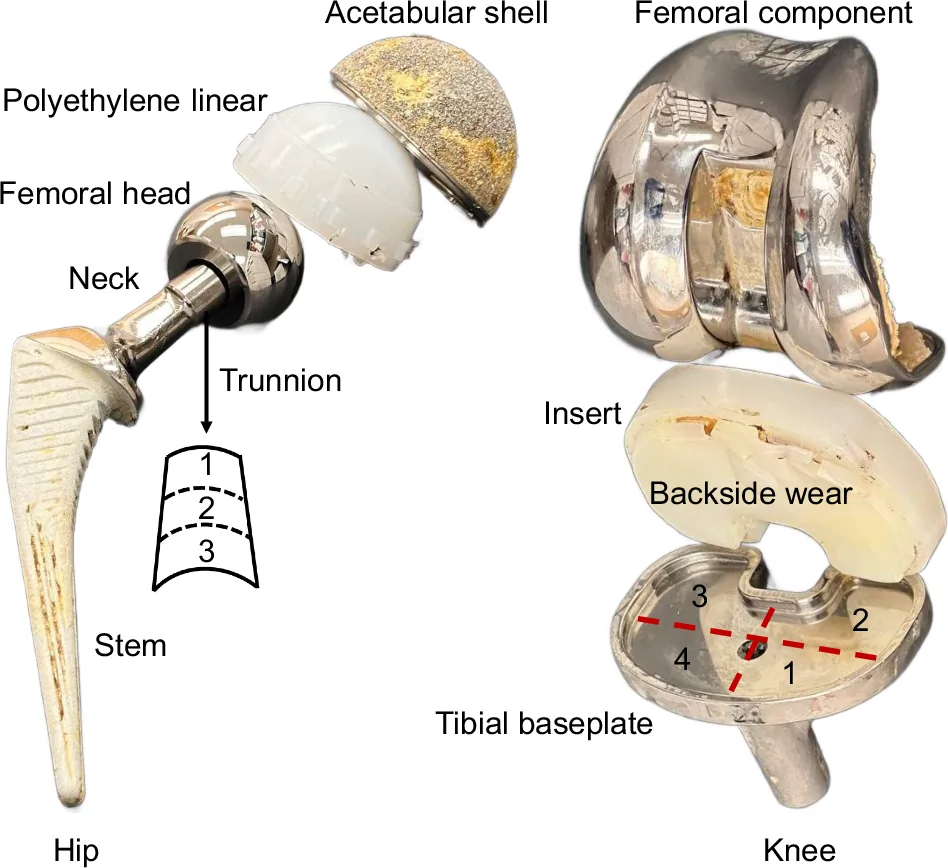
Total hip and knee replacements. Illustration of the investigated artificial hip and knee joint components.

In total, 254 implants, comprising 136 retrieved hips and 118 retrieved knees (made of stainless steel, Ti, and CoCrMo alloys), were explanted from 2017-12-01 to 2020-11-30 and selected after Western University Health Research Ethics Board approval (WREM 118262) for implant retrieval analysis and patient information review. The requirement for informed consent was waived by the ethics board due to the impracticability and impossibility of contacting all previously discharged patients. This study was conducted in accordance with the principles of the World Medical Association’s Declaration of Helsinki. These implants were sterilized by soaking in 10% neutral-buffered formalin for >24 h. The formalin was subsequently neutralized using Formalex solution, and the implant was bathed in a solution of hot water and Extran (a detergent). Loose tissues were scrubbed from the implant with a soft nylon brush. For retrieval analysis, the trunnion of the hip and the superior surface of the tibial component of the knee were studied (Fig. 8). Due to missing patient information, some implants were excluded. 135 hip and 106 knee implants, totaling 241 implants, were included in the final analysis. Figure S1 lists all the patient and implant factors that were collected for this study. Detailed information about the patient and implant factors is provided in the Supporting Information (SI).

### Optical microscopy (OM)

A digital microscope (Olympus Corporation, Tokyo, Japan, model number DSX1000) was used at 20× magnification for the trunnion parts of the hip implants and at 1.4× magnification for the knee implants. The image of the tibial component was stitched together from section images with 20% overlay in the Olympus software. Using the standardized procedure developed by Goldberg et al.^40^, the trunnion part of hip implants was analyzed and given a damage score based on three areas on the surface, as shown in Fig. 8 (1: apex, 2: central, and 3: base). To damage score the tibial baseplates, the tibial component was divided into four quarters, and the extent of damage was evaluated in each quadrant (Fig. 8). In this procedure, the degree of observed damage was similarly quantified, and damage scores from 1 to 4 were assigned for each implant. The scoring criteria for the tibial and the trunnion parts are described in Table 1. Two observers assigned all OM scores with a final consensus. Surgery or surgeon-caused defects, if present, were distinctly visible as deep, long scratches and not considered.

### Scanning electron microscopy (SEM)

For surface and elemental distribution investigations, a Hitachi SU3900 large-chamber variable-pressure SEM, coupled with an Oxford ULTIM MAX 65 SDD X-ray analyzer, was utilized at a 10 mm working distance with an accelerating voltage of 15 kV. This instrument was used for 50 knee and 49 hip implants selected from the 241-sample pool. The SEM scores were assigned based on the criteria listed in Table 2. Two observers assigned all SEM scores with a final consensus. Surgery or surgeon-caused defects, if present, were distinctly visible as deep, long scratches and not considered.

### Data analysis

Among the 241 hip and knee implants studied, the mean age of the patients was 68.7 years (ranging from 32.7 to 99.5 years). Slightly more than half of the implants were retrieved from women (53.9%, *n* = 130). The average implantation length was 5.6 years (ranged from 0.03 to 30.2 years). Statistical analysis was conducted by IBM^®^ SPSS^®^ Statistics, version 26. All hypothesis tests were conducted at a 0.05 significance level. In the results section, we present our analysis results using the following notation: Spearman’s rho correlation by *ρ*, Wilcoxon-Mann-Whitney test statistic by *U* (along with the corresponding *Z* value), Kruskal–Wallis test statistic by *H*, likelihood ratio test statistic for ordinal regression by *T* comparing the null model with the proposed single covariate model, and Pearson Chi-squared test statistic by *χ*^2^. Additionally, Fig. S2 shows the binning method used for input data analysis in SPSS. Detailed information on the data analysis is provided in the SI. The results of all correlations between all implant/patient factors and damage scores (SEM and OM) of either hip or knee implants are listed in Tables S1 and S2.

## Supplementary information

Supplementary Information

## Data Availability

The data that support the findings of this study are available on request from the corresponding author. The data are not publicly available due to ethical restrictions.

## References

[1] Karlson, E. W. et al. Total hip replacement due to osteoarthritis: the importance of age, obesity, and other modifiable risk factors. *Am. J. Med.* **114**, 93–98 (2003). 12586227 10.1016/s0002-9343(02)01447-x

[2] Midgley, J. Osteoarthritis and obesity; conservative management, multi-morbidity, surgery and the implications of restricted access to knee or hip replacement: a literature review. *Int. J. Orthop. Trauma Nurs.* **40**, 100840 (2021). 33461941 10.1016/j.ijotn.2020.100840

[3] Jin, W. & Chu, P. K. Orthopedic implants. *Encycl. Biomed. Eng.* **1–3**, 425–439 (2019).

[4] Niinomi, M., Nakai, M. & Hieda, J. Development of new metallic alloys for biomedical applications. *Acta Biomater.* **8**, 3888–3903 (2012). 22765961 10.1016/j.actbio.2012.06.037

[5] Manam, N. S. et al. Study of corrosion in biocompatible metals for implants: a review. *J. Alloys Compd.* **701**, 698–715 (2017).

[6] Eliaz, N. Corrosion of metallic biomaterials: a review. *Materials* **12**, 407 (2019). 30696087 10.3390/ma12030407 PMC6384782

[7] Shelly, S. et al. Potential neurotoxicity of titanium implants: prospective, in-vivo and in-vitro study. *Biomaterials* **276**, 121039 (2021). 34352627 10.1016/j.biomaterials.2021.121039

[8] Bridges, R. L., Cho, C. S., Beck, M. R., Gessner, B. D. & Tower, S. S. F-18 FDG PET brain imaging in symptomatic arthroprosthetic cobaltism. *Eur. J. Nucl. Med. Mol. Imaging* **47**, 1961–1970 (2020). 31863138 10.1007/s00259-019-04648-2 PMC7299907

[9] Matusiewicz, H. Potential release of in vivo trace metals from metallic medical implants in the human body: from ions to nanoparticles—a systematic analytical review. *Acta Biomater.* **10**, 2379–2403 (2014). 24565531 10.1016/j.actbio.2014.02.027

[10] Pourbaix, M. Electrochemical corrosion of metallic biomaterials. *Biomaterials* **5**, 122–134 (1984). 6375748 10.1016/0142-9612(84)90046-2

[11] Kocijan, A., Milošev, I. & Pihlar, B. The influence of complexing agent and proteins on the corrosion of stainless steels and their metal components. *J. Mater. Sci. Mater. Med.* **14**, 69–77 (2003). 15348541 10.1023/a:1021505621388

[12] Wittrock, A. et al. Protein-metal interactions due to fretting corrosion at the taper junction of hip implants: an in vitro investigation using Raman spectroscopy. *Acta Biomater.* **189**, 621–632 (2024). 10.1016/j.actbio.2024.10.006 39393659

[13] Liang, M. et al. Materials Science & Engineering C Influences of aggressive ions in human plasma on the corrosion behavior of AZ80 magnesium alloy. *Mater. Sci. Eng. C* **119**, 111521 (2021). 10.1016/j.msec.2020.111521 33321608

[14] Kashi, A. & Saha, S. Failure mechanisms of medical implants and their effects on outcomes. In *Biointegration of Medical Implant Materials* 407–432 (Woodhead Publishing, 2019).

[15] Mitchell, A. & Shrotriya, P. Mechanical load-assisted dissolution of metallic implant surfaces: influence of contact loads and surface stress state. *Acta Biomater.* **4**, 296–304 (2008). 17901005 10.1016/j.actbio.2007.08.004

[16] Bermúdez-Castañeda, A., Igual-Muñoz, A. & Mischler, S. A crevice corrosion model for biomedical trunnion geometries and surfaces feature. *Materials* **14**, 1–17 (2021). 10.3390/ma14041005 PMC7924358 33672713

[17] Sotniczuk, A. Corrosion resistance of β-phase titanium alloys under simulated inflammatory conditions: exploring the relevance of biocompatible alloying elements. *Corros. Sci.* **220**, 111381 (2023).

[18] Hao, Y. et al. Improved corrosion behaviour of electron beam melted Ti-6Al–4V alloy in phosphate buffered saline. *Corros. Sci.* **123**, 289–296 (2017).

[19] Wauthle, R. et al. Additively manufactured porous tantalum implants. *Acta Biomater.* **14**, 217–225 (2015). 25500631 10.1016/j.actbio.2014.12.003

[20] Boyer, B. et al. What are the influencing factors on hip and knee arthroplasty survival? Prospective cohort study on 63619 arthroplasties. *Orthop. Traumatol. Surg. Res.* **105**, 1251–1256 (2019). 31615748 10.1016/j.otsr.2019.07.020

[21] Codirenzi, A. M., Lanting, B. A. & Teeter, M. G. What patient and implant factors affect trunnionosis severity? An implant retrieval analysis of 664 femoral stems. *J. Arthroplasty* **38**, 376–382 (2023). 36084756 10.1016/j.arth.2022.08.023

[22] Kokko, M. A., Abdel, M. P., Berry, D. J., Butler, R. D. & Van Citters, D. W. A retrieval analysis perspective on revision for infection. *Arthroplasty Today* **5**, 362–370 (2019). 31516983 10.1016/j.artd.2019.03.007 PMC6728442

[23] Avery, D. et al. Obesity prolongs the pro-inflammatory response and attenuates bone healing on titanium implants. *Acta Biomater.* **192**, 473–486 (2024). 39586347 10.1016/j.actbio.2024.11.040 PMC11735295

[24] Gilbert, J. L., Buckley, C. A. & Jacobs, J. J. In vivo corrosion of modular hip prosthesis components in mixed and similar metal combinations. The effect of crevice, stress, motion, and alloy coupling. *J. Biomed. Mater. Res.* **27**, 1533–1544 (1993). 8113241 10.1002/jbm.820271210

[25] Hall, D. J., Pourzal, R. & Jacobs, J. J. What surgeons need to know about adverse local tissue reaction in total hip arthroplasty. *J. Arthroplasty* **35**, S55–S59 (2020). 32005621 10.1016/j.arth.2020.01.016 PMC7239747

[26] Vasconcelos, D. M., Santos, S. G., Lamghari, M. & Barbosa, M. A. The two faces of metal ions: from implants rejection to tissue repair/regeneration. *Biomaterials* **84**, 262–275 (2016). 26851391 10.1016/j.biomaterials.2016.01.046

[27] Martin, A. J., Seagers, K. A. & Van Citters, D. W. Assessment of corrosion, fretting, and material loss of retrieved modular total knee arthroplasties. *J. Arthroplasty* **32**, 2279–2284 (2017). 28343824 10.1016/j.arth.2017.02.047

[28] Del Balso, C., Teeter, M. G., Tan, S. C., Lanting, B. A. & Howard, J. L. Taperosis: Does head length affect fretting and corrosion in total hip arthroplasty?. *Bone Jt. J.* **97-B**, 911–916 (2015). 10.1302/0301-620X.97B7.35149 26130345

[29] Jauch, S. Y., Huber, G., Haschke, H., Sellenschloh, K. & Morlock, M. M. Design parameters and the material coupling are decisive for the micromotion magnitude at the stem-neck interface of bi-modular hip implants. *Med. Eng. Phys.* **36**, 300–307 (2014). 24332894 10.1016/j.medengphy.2013.11.009

[30] Ghadirinejad, K. et al. Fretting wear and corrosion-related risk factors in total hip replacement: a literature review on implant retrieval studies and national joint replacement registry reports. *Prosthesis* **5**, 774–791 (2023).

[31] Milovanovic, P. et al. Age- and sex-specific bone structure patterns portend bone fragility in radii and tibiae in relation to osteodensitometry: a high-resolution peripheral quantitative computed tomography study in 385 individuals. *J. Gerontol. Ser. A Biol. Sci. Med. Sci.* **70**, 1269–1275 (2014). 10.1093/gerona/glv052 25934995

[32] Arteaga, A. et al. Diabetes as a risk factor for orthopedic implant surface performance: a retrieval and in vitro study. *J. Bio Tribocorrosion* **7**, 1–15 (2021). 10.1007/s40735-021-00486-8 PMC8211117 34150468

[33] Lin, Z. H., Chang, H. C., Wu, Y. L. & Gau, S. Y. Increased risk of new-onset rheumatoid arthritis among osteoarthritis patients received total knee arthroplasty: a global federated health network analysis. *Int. J. Med. Sci.* **21**, 994–1002 (2024). 38774753 10.7150/ijms.93457 PMC11103392

[34] Gilbert, J. L. Corrosion in the human body: metallic implants in the complex body environment. *Corrosion* **73**, 1478–1495 (2017).

[35] Higgs, G. B. et al. Does taper size have an effect on taper damage in retrieved metal-on-polyethylene total hip devices?. *J. Arthroplasty* **31**, 277–281 (2016). 27460298 10.1016/j.arth.2016.06.053

[36] Arnholt, C. M. et al. Corrosion damage and wear mechanisms in long-term retrieved CoCr femoral components for total knee arthroplasty. *J. Arthroplasty* **31**, 2900–2906 (2016). 27426028 10.1016/j.arth.2016.05.006 PMC5107165

[37] Kurtz, M. A. et al. Tibial baseplate microstructure governs high cycle fatigue fracture in vivo. *J. Biomed. Mater. Res. Part B Appl. Biomater.* **112**, 1–14 (2024). 10.1002/jbm.b.35507 39570093

[38] Aslani, S. et al. Crevice corrosion degrades metaphyseal sleeves following total knee arthroplasty: a retrieval study. *J. Arthroplasty* **40**, 3324–3334 (2025). 40480330 10.1016/j.arth.2025.05.118

[39] Li, S. et al. Assessment of backside wear from the analysis of 55 retrieved tibial inserts. *Clin. Orthop. Relat. Res.* **404**, 75–82 (2002). 10.1097/00003086-200211000-00013 12439241

[40] Goldberg, J. R. et al. A multicenter retrieval study of the taper interfaces of modular hip prostheses. *Clin. Orthop. Relat. Res.* **401**, 149–161 (2002). 10.1097/00003086-200208000-00018 12151892

[41] Atapour, M. et al. Influence of proteins and building direction on the corrosion and tribocorrosion of CoCrMo fabricated by laser powder bed fusion. *ACS Biomater. Sci. Eng.* **10**, 2880–2893 (2024). 38630940 10.1021/acsbiomaterials.3c01165

[42] Yang, S. et al. A preliminary experimental investigation on the biotribocorrosion of a metal-on-polyethylene hip prosthesis in a hip simulator. *Friction* **11**, 1094–1106 (2023).

[43] Swaminathan, V. & Gilbert, J. L. Fretting corrosion of CoCrMo and Ti6Al4V interfaces. *Biomaterials* **33**, 5487–5503 (2012). 22575833 10.1016/j.biomaterials.2012.04.015

[44] Siljander, M. P. et al. Fretting and corrosion damage in retrieved metal-on-polyethylene modular total hip arthroplasty systems: What is the importance of femoral head size?. *J. Arthroplasty* **33**, 931–938 (2018). 29113756 10.1016/j.arth.2017.10.010

[45] Gilbert, J. L. et al. Direct in vivo inflammatory cell-induced corrosion of CoCrMo alloy orthopedic implant surfaces. *J. Biomed. Mater. Res. Part A* **103**, 211–223 (2015). 10.1002/jbm.a.35165 PMC4162871 24619511

[46] Bruggink, C., Gerards, R. & Nijs, A. Gross trunnion failure in an elderly obese patient presenting 10 years after total hip arthroplasty with a cobalt chromium femoral head: a case report. *Int. J. Surg. Case Rep.* **118**, 109525 (2024). 38555830 10.1016/j.ijscr.2024.109525 PMC10987315

[47] Collier, J. P., Surprenant, V. A., Jensen, R. E., Mayor, M. B. & Surprenant, H. P. Corrosion between the components of modular femoral hip prostheses. *J. Bone Jt. Surg. Ser. B* **74**, 511–517 (1992). 10.1302/0301-620X.74B4.1624507 1624507

[48] Wilson, C. J., Theodoulou, A., Damarell, R. A. & Krishnan, J. Knee instability as the primary cause of failure following Total Knee Arthroplasty (TKA): a systematic review on the patient, surgical and implant characteristics of revised TKA patients. *Knee* **24**, 1271–1281 (2017). 28970123 10.1016/j.knee.2017.08.060

[49] Sisko, Z. W. et al. Current total knee designs: Does baseplate roughness or locking mechanism design affect polyethylene backside wear?. *Clin. Orthop. Relat. Res.* **475**, 2970–2980 (2017). 28905208 10.1007/s11999-017-5494-3 PMC5670066

[50] Rao, A. R., Engh, G. A., Collier, M. B. & Lounici, S. Tibial interface wear in retrieved total knee components and correlations with modular insert motion. *J. Bone Jt. Surg.* **84**, 1849–1855 (2002). 10.2106/00004623-200210000-00017 12377918

[51] Jeyalakshmi, P. & Ramkumar, P. Failure mechanisms and influencing factors on orthopaedic knee implant failure—a comprehensive review. *Proc. Inst. Mech. Eng. Part J J. Eng. Tribol.* **239**, 263–282 (2025).

[52] Maag, C., Metcalfe, A., Cracaoanu, I., Wise, C. & Auger, D. D. The development of simulator testing for total knee replacements. *Biosurface and Biotribology* **7**, 70–82 (2021).

[53] Brien, S. O., Luo, Y., Wu, C., Petrak, M. & Bohm, E. Prediction of backside micromotion in total knee replacements by finite element simulation. *Proc. Inst. Mech. Eng. H* **226**, 235–245 (2012). 10.1177/0954411911435593 22558838

[54] Zhou, Y., Harries, D. & Stoney, J. D. A polished cobalt-chrome baseplate is not associated with a lower revision rate than matt titanium in a single total knee arthroplasty implant system with identical baseplate design. *J. Arthroplasty* **39**, 896–903 (2024). 37852451 10.1016/j.arth.2023.10.003

[55] Martin, J. R., Watts, C. D., Levy, D. L. & Kim, R. H. Medial tibial stress shielding: a limitation of cobalt chromium tibial baseplates. *J. Arthroplasty* **32**, 558–562 (2017). 27593733 10.1016/j.arth.2016.07.027

[56] Berend, M. E., Ritter, M. A., Hyldahl, H. C., Meding, J. B. & Redelman, R. Implant migration and failure in total knee arthroplasty is related to body mass index and tibial component size. *J. Arthroplasty* **23**, 104–109 (2008). 18722310 10.1016/j.arth.2008.05.020

[57] Galas, A., Banci, L. & Innocenti, B. The effects of different femoral component materials on bone and implant response in total knee arthroplasty: a finite element analysis. *Materials* **16**, 5605 (2023). 37629896 10.3390/ma16165605 PMC10456576

[58] Antonelli, B. & Chen, A. F. Reducing the risk of infection after total joint arthroplasty: preoperative optimization. *Arthroplasty* **1**, 1–13 (2019). 35240760 10.1186/s42836-019-0003-7 PMC8787890

[59] Triantafyllopoulos, G., Stundner, O., Memtsoudis, S. & Poultsides, L. A. Patient, surgery, and hospital related risk factors for surgical site infections following total hip arthroplasty. *Sci. World J*. **2015**, 979560 (2015). 10.1155/2015/979560 PMC4446513 26075298

[60] Matin, S. et al. A bio-tribocorrosion comparison between additively manufactured and forged Ti6Al4V parts. *Addit. Manuf. Lett.* **7**, 100156 (2023).

[61] Li, C. Y., Cheong Chung, K. J. N., Ali, O. M. E., Chung, N. D. H. & Li, C. H. Literature review of the causes of pain following total knee replacement surgery: prosthesis, inflammation and arthrofibrosis. *EFORT Open Rev.* **5**, 534–543 (2020). 33072405 10.1302/2058-5241.5.200031 PMC7528670

[62] Emara, A. K. et al. Femoral stem cementation in hip arthroplasty: the know-how of a “lost” art. *Curr. Rev. Musculoskelet. Med.* **14**, 47–59 (2021). 33453016 10.1007/s12178-020-09681-5 PMC7930165

[63] Soydan, A. M., Yıldız, D. S., Bilgen, Ö. F. & Uyanık, M. S. Total hip arthroplasty: effect of head size, offset gap, offset angle, trunnion length and fixation on trunnionosis of hip implant. *Int. J. Numer. Method. Biomed. Eng*. **41**, e70106 (2025). 10.1002/cnm.70106 41131926

[64] Savio, D. & Bagno, A. When the total hip replacement fails: a review on the stress-shielding effect. *Processes* **10**, 612 (2022).

[65] Bormann, T., Kretzer, J. P., Jaeger, S. & Lohmann, C. H. Is taper corrosion in modular revision hip stem junctions associated with patient or implant specific factors? A retrieval analysis. *J. Mech. Behav. Biomed. Mater.* **150**, 106326 (2024). 38141361 10.1016/j.jmbbm.2023.106326

