# Supplementary material for: Corrosion and wear damage estimations for retrieved knee and hip implants
Source: Npj Mater Degrad. 2026 Jun 3;10(1):110. doi: 10.1038/s41529-026-00821-9 (PMC13541609; doi:10.1038/s41529-026-00821-9)
Supplement: Supplementary file 1 [file 41529_2026_821_MOESM1_ESM.pdf]

Supplementary information

Corrosion and wear damage estimations for retrieved knee and hip implants

Saman Nikpour[a,d,*], Anastasia Codirenzi[b], Christine Hoskin[a], Ubong Eduok[a], Brent Lanting[c,d], Matthew G. Teeter[b,d], Camila P. E. de Souza[e], Yolanda S. Hedberg[a,d,f,*]
[a]*Department of Chemistry, The University of Western Ontario, London, Ontario, Canada*
[b]*Department of Medical Biophysics, Schulich School of Medicine and Dentistry, The University of Western Ontario, London, Canada*
[c]*Department of Surgery, Schulich School of Medicine and Dentistry, The University of Western Ontario, London, Canada.*
[d]*Department of Orthopaedic Surgery, London Health Sciences Centre, University Hospital, London, Ontario, Canada*
[e]*Department of Statistical and Actuarial Sciences, The University of Western Ontario, London, Ontario, Canada*
[f]*Surface Science Western, The University of Western Ontario, London, Ontario N6G 0J3, Canada*

*Corresponding authors: Saman Nikpour and Yolanda Hedberg, Department of Chemistry, The University of Western Ontario, N6A 5B7 London, Canada. snikpou3@uwo.ca; yhedberg@uwo.ca.

Patient information

- Sex (f/m)
- Age (year)
- Height (cm)
- Weight (kg)
- BMI (kg/m^2)
- Implantation Length (years of implantation)
- Number of Revisions
- Diabetes (type I, type II, none)
- Inflammatory Arthritis
- Pain
- Infection (prior or at surgery, 6 weeks after surgery, and due to other surgeries)

General implant information

- Type (hip or knee)
- Loosening (aseptic, septic, none)
- Instability
- Manufacturer
- Material
- Design

Hip implant information

- Stem Model
- Stem Material
- Stem Offset (standard, high)
- Stem Cemented
- Stem Collared
- Head Material
- Head Model
- Head Size (mm)
- Head Offset (mm)
- Implant System

Knee implant information

- Femoral size
- Femoral A/P and M/L (mm)
- Femoral Material
- Femoral Cemented
- Tibial Size
- Tibial A/P and M/L (mm)
- Tibial Material
- Implant System

Fig. S1. Patient and implant parameters that were collected for data analysis.

Patient information, including sex, age, weight, height, body mass index (BMI), implantation length (how many years the implant was in the body before it was taken out), having infection (distinguishing if it was prior to/at surgery, 6 weeks after surgery, and due to other surgeries), diabetes (distinguishing type I and II), pain, and inflammatory arthritis, was obtained from the Chart review (London Health Sciences Center). In addition, implant information, including material, manufacturer, and design (femoral head size, tibial size, system, bone cement, offset, collar, etc.), loosening (distinguishing septic and aseptic loosening), and instability, was collected from the implant retrieval laboratory catalogue.

Implant System	
0	No data
1	NexGen
2	Attune
3	Triathlon
4	Sigma
5	Gen II
6	Accolade
7	Accolade I
8	AML
9	Corail
10	CPS
11	Echelon
12	Exeter
13	LDH
14	ML taper
15	Omnifit
16	Polar
17	Prodigy
18	Reclaim
19	Redapt
20	Rejuvenate
21	Restoration
22	Securefit
23	SMF
24	Solution MMA
25	Synergy
26	Synergy II
27	Spectrum
28	Spectron
29	Spectron EF
30	Taperloc
31	TMZF
32	Versys
33	ML Taper Triology
34	Summit

Implant Manufacturer	
0	No data
1	Zimmer
2	Depuy
3	Stryker
4	S&N
5	Biomet
6	Microport
7	Biomet + Zimmer
8	DePuy + Stryker
9	DePuy + Zimmer
10	J&J + DePuy
11	J&J + DePuy + S&N
12	S&N + DePuy
13	Stryker + S&N
14	Zimmer + Triology
15	Zimmer + DePuy

Age	
0	No data
1	30-40 years
2	40-50 years
3	50-60 years
4	60-70 years
5	70-80 years
6	80-90 years
7	90-100 years
8	>100 years

Implant Type	
1	Knee
2	Hip

Sex	
0	No data
1	F
2	M

Height	
0	No data
1	130-140 cm
2	140-150 cm
3	150-160 cm
4	160-170 cm
5	170-180 cm
6	180-190 cm
7	190-200 cm
8	>200 cm

Weight	
0	No data
1	40-60 kg
2	60-80 kg
3	80-100 kg
4	100-120 kg
5	120-140 kg
6	140-160 kg
7	>160 kg

Implantation Length	
0	No data
1	<1 year
2	1-2 years
3	2-3 years
4	3-4 years
5	4-5 years
6	5-10 years
7	10-15 years
8	15-20 years
9	>20 years

Pain	
0	No data
1	No
2	Yes

BMI Threshold	
0	No data
1	Severe Thinness (<16)
2	Moderate Thinness (16-17)
3	Mild Thinness (17-18.5)
4	Healthy (18.5-25)
5	Overweight (25-30)
6	Obese: Class I (30-35)
7	Obese: Class II (35-40)
8	Obese: Class III (>40)

Infection prior/at surgery	
0	No data
1	No
2	Yes

Infection after surgery	
0	No data
1	No
2	Yes

Infection due to other surgeries	
0	No data
1	No
2	Yes

Implant Material	
0	No data
1	CoCrMo
2	Ti
3	Ceramic
4	CoCr

Diabetes	
0	No data
1	No
2	Type 1
3	Type 2

Loosening	
0	No data
1	None
2	Aseptic
3	Septic

Arthritis	
0	No data
1	No
2	Yes

Instability	
0	No data
1	No
2	Yes

Bone Cement	
0	No data
1	No
2	Yes

Hip Stem Offset	
0	No data
1	Standard offset
2	High offset

Hip Stem Collared	
0	No data
1	No
2	Yes

Damage Score (SEM or OM)	
0	No data
1	None
2	Mild
3	Moderate
4	Severe

Fig. S2. Categories and binning used for data analysis. All misspellings were corrected to clean the data before categorization and binning.

Initially, the correlation between ordinal variables such as SEM *vs.* OM scores or binned variables was evaluated by Spearman's rho correlation. Then, the ordinal regression was employed between an ordinal damage/corrosion score (SEM or OM) and each set of continuous variables, such as implantation length, age, weight, and height to find any possible influence on the damage of the implants.

To compare damage scores (SEM or OM) and implantation length across different levels of binary categorical variables (implant type, infection, arthritis, and bone cement), the non-parametric Wilcoxon-Mann-Whitney test was conducted. In the case of a categorical variable with more than two levels (implant systems), the non-parametric Kruskal-Wallis's test was employed. The association between two categorical variables, such as bone cementation status and having arthritis, was analyzed using the Pearson chi-square test. To control the effect of a continuous variable (such as implantation length) and a categorical variable (such as sex) in the damage scores while studying the effect of other factors, the ordinal regression was used, and the P value is reported.

Table S1. Summary of correlations between implant/patient factors and damage scores (SEM and OM) of knee tibial baseplates.

**Knee implant**	SEM	OM
**Patient Factors**		
Age	$T = 0.422$, $P = 0.516$	$T = 0.491$, $P = 0.483$
Age Binning	$\rho = -0.106$, $P = 0.462$	$\rho = -0.044$, $P = 0.656$
Height	$T = 0.716$, $P = 0.398$	$T = 0.296$, $P = 0.587$
Height Binning	$\rho = -0.120$, $P = 0.408$	$\rho = -0.073$, $P = 0.463$
Weight	$T = 0.016$, $P = 0.900$	$T = 0.027$, $P = 0.871$
Weight Binning	$\rho = -0.061$, $P = 0.675$	$\rho = 0.033$, $P = 0.739$
Implantation length	$T = 3.057$, $P = 0.080$	$T = 2.548$, $P = 0.110$
Implantation length Binning	$\rho = 0.265$, $P = 0.104$	$\rho = 0.097$, $P = 0.361$
BMI	$T = 0.453$, $P = 0.501$	$T = 0.118$, $P = 0.731$
BMI Binnig	$\rho = 0.049$, $P = 0.734$	$\rho = 0.022$, $P = 0.823$
Sex	$U = 273$, $P = 0.849$	$U = 1227.5$, $P = 0.316$
Revision number	$T = 0.493$, $P = 0.483$	$T = 2.240$, $P = 0.135$
Arthritis	$U = 123$, $P = 0.244$	$U = 842.5$, $P = 0.878$
Diabetes	$H = 0.051$, $P = 0.821$	$H = 1.158$, $P = 0.560$
Loosening	$H = 1.297$, $P = 0.523$	$H = 2.454$, $P = 0.293$
Instability	$U = 288$, $P = 1.000$	$U = 1253.5$, $P = 0.869$
Pain	$U = 17$, $P = 0.519$	$U = 40.5$, $P = 0.671$
Infection prior to/at surgery	$U = 232.5$, $P = 0.430$	$U = 1089$, $P = 0.981$
Infection 6 weeks after surgery	**$U = 10.5$, $P = 0.021$**	$U = 200$, $P = 0.971$
Infection due to other surgery	$U = 62$, $P = 0.183$	$U = 344$, $P = 0.534$
**Implant Factors**		
Implant manufacturer	$H = 5.44$, $P = 0.142$	**$H = 49.63$, $P < 0.001$**
Implant system	$H = 9.191$, $P = 0.057$	**$H = 50.36$, $P < 0.001$**
Femoral size	$H = 6.438$, $P = 0.490$	$H = 7.090$, $P = 0.527$
Femoral material	N/A	N/A
Femoral A/P	$T = 0.187$, $P = 0.665$	$T = 2.042$, $P = 0.153$

Femoral M/L	$T = 0.146$, $P = 0.703$	$T = 0.472$, $P = 0.492$
Femoral cemented	N/A	N/A
Tibial size	$T = 0.042$, $P = 0.838$	$T = 0.025$, $P = 0.874$
Tibial cemented	N/A	N/A
Tibial material	N/A	N/A

Table S2. Summary of correlations between implant/patient factors and damage scores (SEM and OM) of hip trunnion.

**Hip implant**	SEM	OM
**Patient Factors**		
Age	$T = 2.248$, $P = 0.134$	$T = 0.097$, $P = 0.755$
Age Binning	$\rho = -0.256$, $P = 0.076$	$\rho = -0.005$, $P = 0.956$
Height	$T = 3.360$, $P = 0.067$	$T = 0.614$, $P = 0.433$
Height Binning	$\rho = 0.260$, $P = 0.071$	$\rho = -0.035$, $P = 0.683$
Weight	$T = 3.290$, $P = 0.070$	$T = 0.166$, $P = 0.683$
Weight Binning	**$\rho = 0.331$, $P = 0.020$**	$\rho = -0.049$, $P = 0.572$
Implantation length	$T = 2.003$, $P = 0.157$	$T = 1.918$, $P = 0.166$
Implantation length Binning	$\rho = -0.221$, $P = 0.182$	$\rho = 0.027$, $P = 0.777$
BMI	$T = 0.453$, $P = 0.501$	$T = 0.068$, $P = 0.794$
BMI Binnig	$\rho = 0.798$, $P = 0.372$	$\rho = 0.035$, $P = 0.688$
Sex	$U = 256.5$, $P = 0.395$	$U = 2249.5$, $P = 0.898$
Revision number	$T = 0.677$, $P = 0.411$	$T = 0.070$, $P = 0.791$
Arthritis	**$U = 83$, $P = 0.022$**	$U = 1155$, $P = 0.578$
Diabetes	$H = 1.366$, $P = 0.505$	$H = 1.482$, $P = 0.477$
Loosening	$H = 2.445$, $P = 0.294$	$H = 0.265$, $P = 0.876$
Instability	$U = 65.5$, $P = 0.879$	$U = 826.5$, $P = 0.585$
Pain	N/A	N/A
Infection prior to/at surgery	**$U = 163$, $P = 0.012$**	$U = 2206.5$, $P = 0.838$
Infection 6 weeks after surgery	$U = 116.5$, $P = 0.465$	$U = 1121$, $P = 0.610$

Infection due to other surgery	$U = 184.5, P = 0.786$	$U = 1460.5, P = 0.090$
**Implant Factors**		
Implant manufacturer	$H = 10.038, P = 0.262$	$H = 13.581, P = 0.481$
Implant system	$H = 16.620, P = 0.342$	$H = 28.990, P = 0.413$
Hip stem material	$H = 1.894, P = 0.388$	$H = 4.083, P = 0.130$
Hip stem offset	$U = 235.5, P = 0.289$	$U = 1584.5, P = 0.492$
Hip stem cemented	$\mathbf{U = 149.5, P = 0.002}$	$U = 1911, P = 0.515$
Hip stem collared	$U = 215.5, P = 0.496$	$U = 1772.5, P = 0.136$
Hip head material	$H = 1.821, P = 0.177$	$H = 0.474, P = 0.491$
Hip head manufacturer	N/A	N/A
Hip head model	$H = 1, P = 0.317$	$H = 1.926, P = 0.749$
Hip head size	$T = 0.135, P = 0.713$	$T = 0.267, P = 0.997$

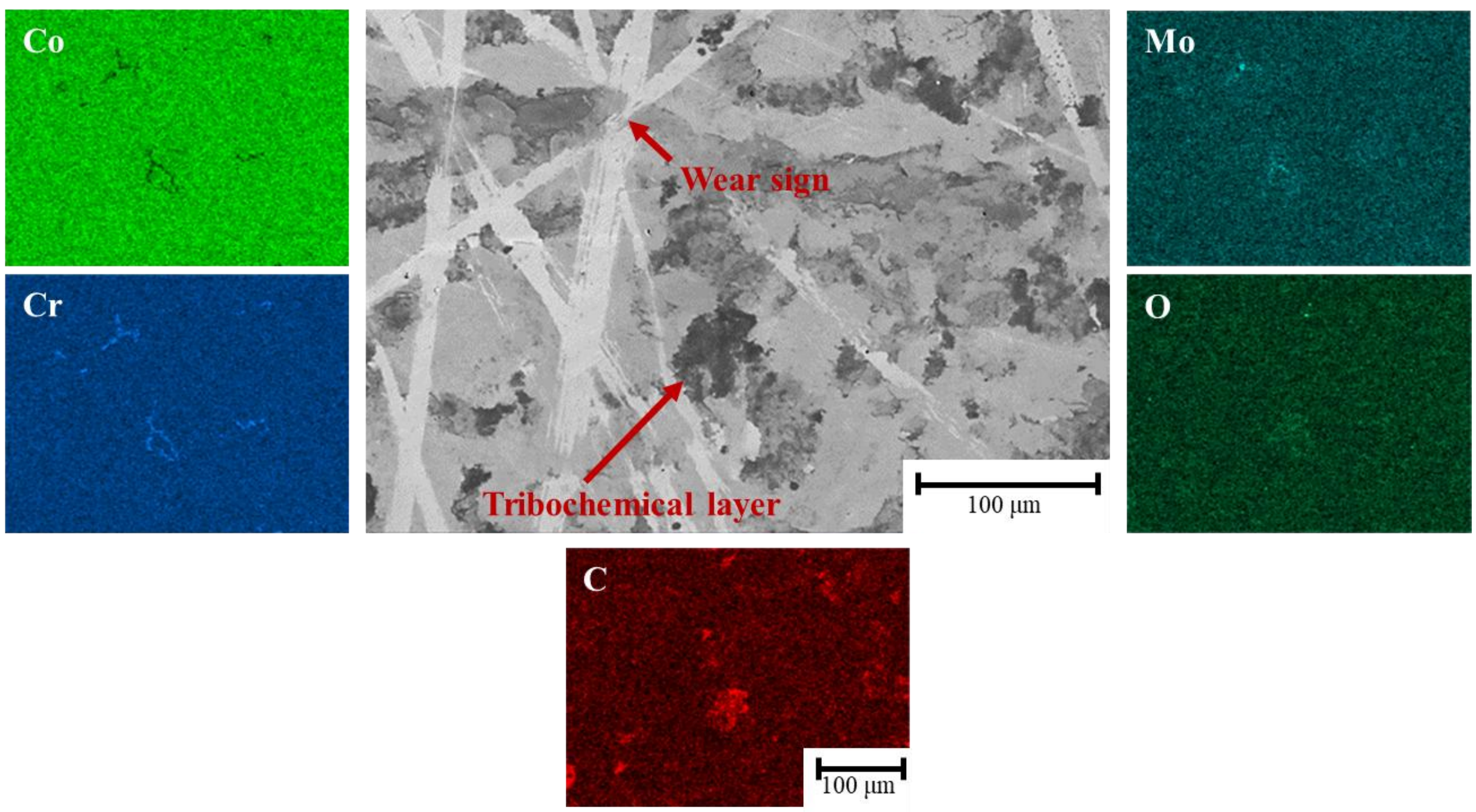

Fig. S3. Example of mechanically assisted corrosion (tribocorrosion) of the tibial part of a knee implant (CoCrMo alloy). The SEM image is placed in the center, and corresponding EDS maps show the elemental distribution of the elements indicated. Wear signs and tribochemical layers are shown by red arrows.

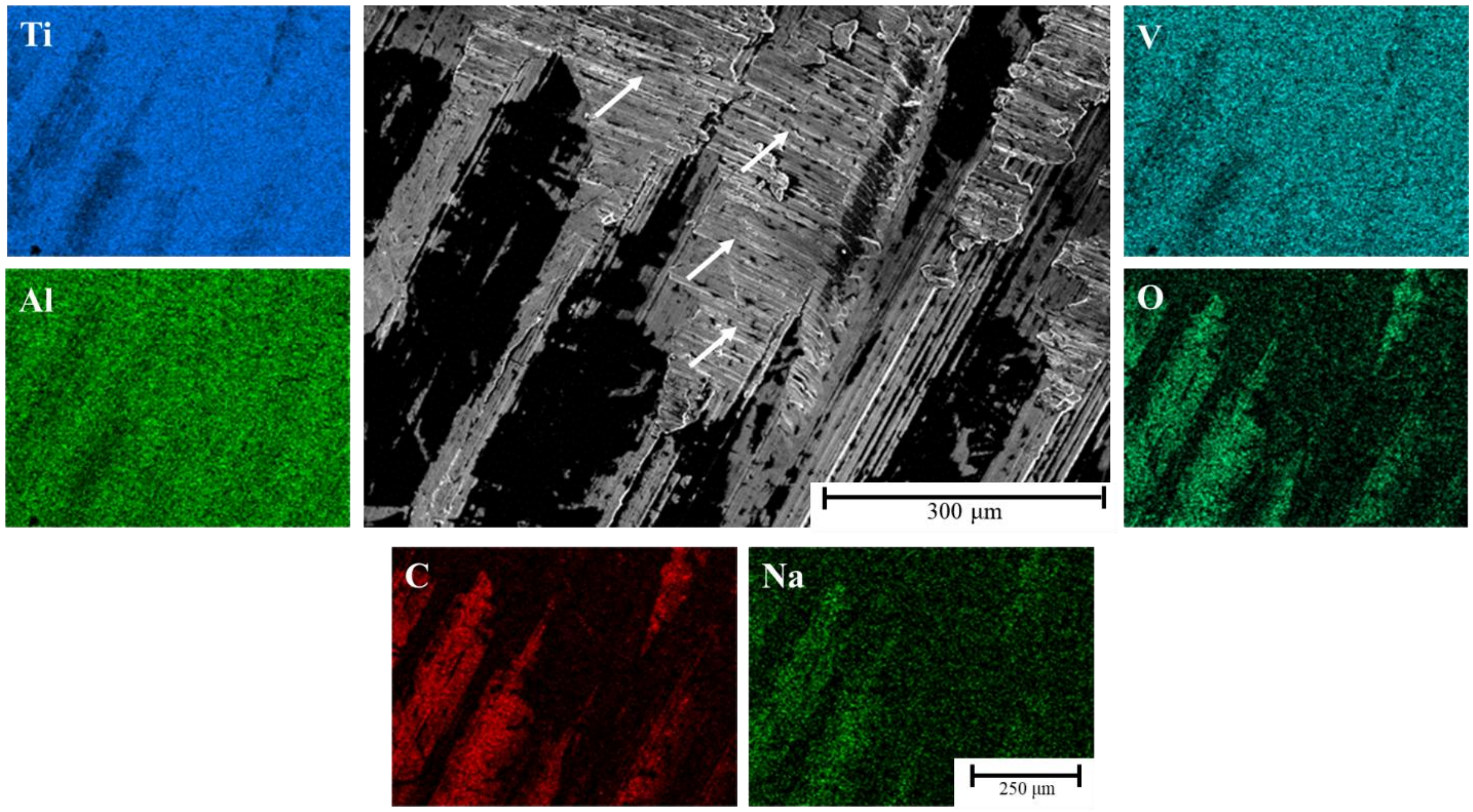

Fig. S4. Example of fretting of the trunnion region of a hip implant (Ti6Al4V alloy). The SEM image is placed in the center, and the corresponding EDS maps show the elemental distribution of the elements indicated. White arrows show fretting lines.

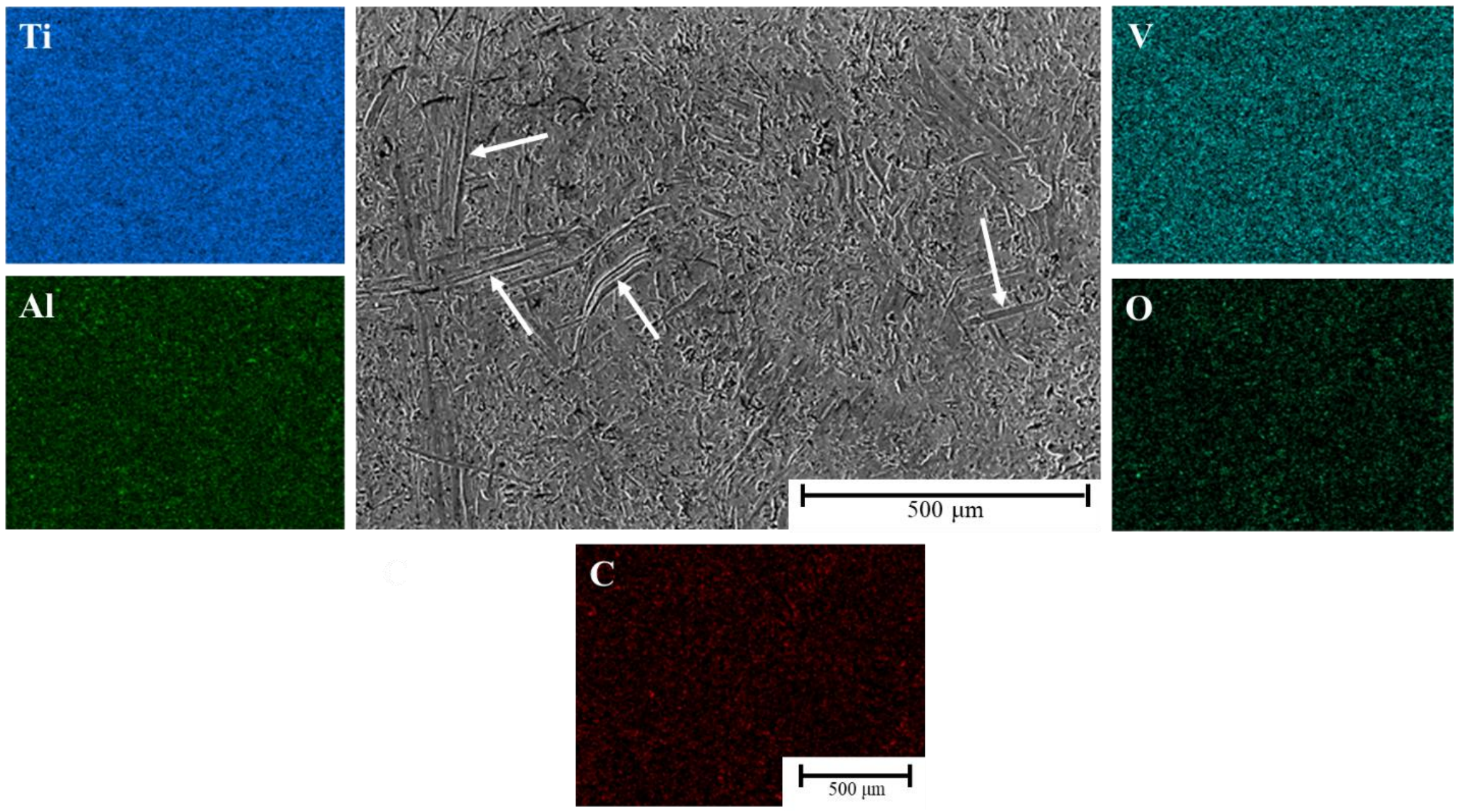

Fig. S5. Example of third-body wear on the surface of the tibial component of a knee implant (Ti6Al4V alloy). The SEM image is placed in the center, and the corresponding EDS maps show the elemental distribution of the elements indicated. White arrows show deep scratches generated by third bodies.

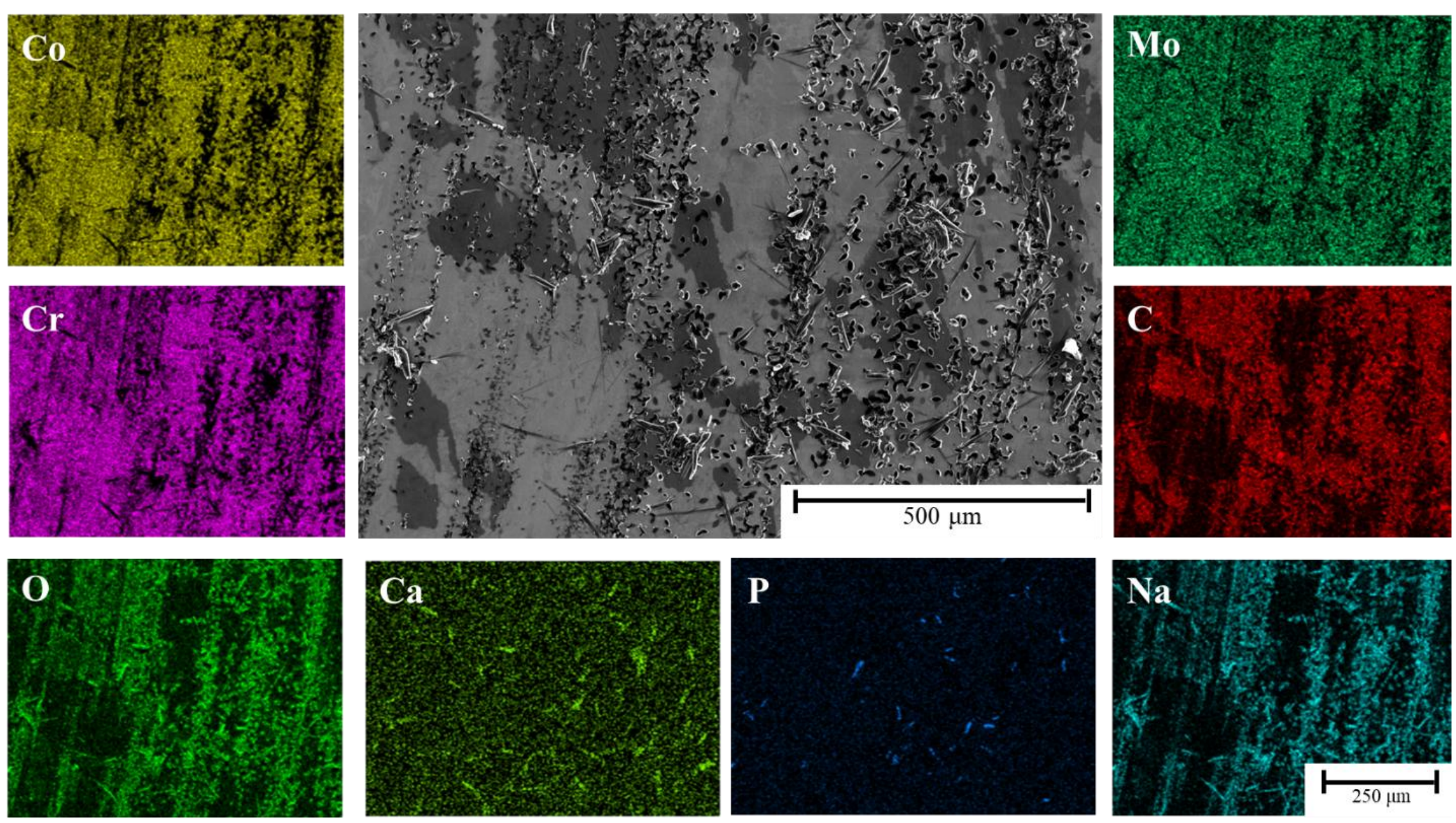

Fig. S6. Example of adsorbed organic species on the surface of the tibial component of a knee implant (CoCrMo alloy). The SEM image is placed in the center, and the corresponding EDS maps show the elemental distribution of the elements indicated.

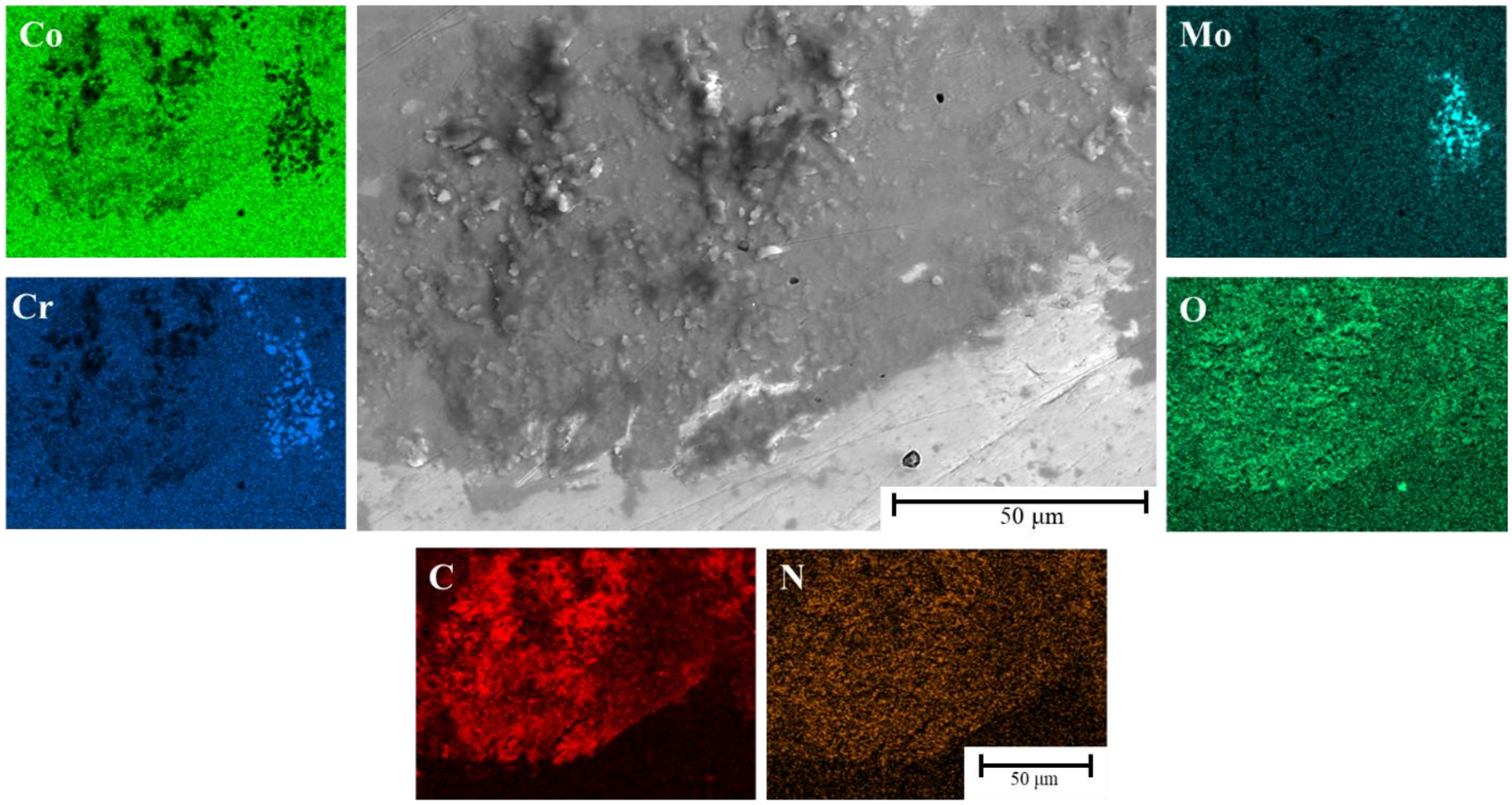

Fig. S7. Example of biofilm corrosion on the surface of the tibial component of a knee implant (CoCrMo alloy). Localized corrosion beneath the biofilm is indicated by a local enrichment of Cr and Mo. The SEM image is placed in the center, and the corresponding EDS maps show the elemental distribution of the elements indicated.

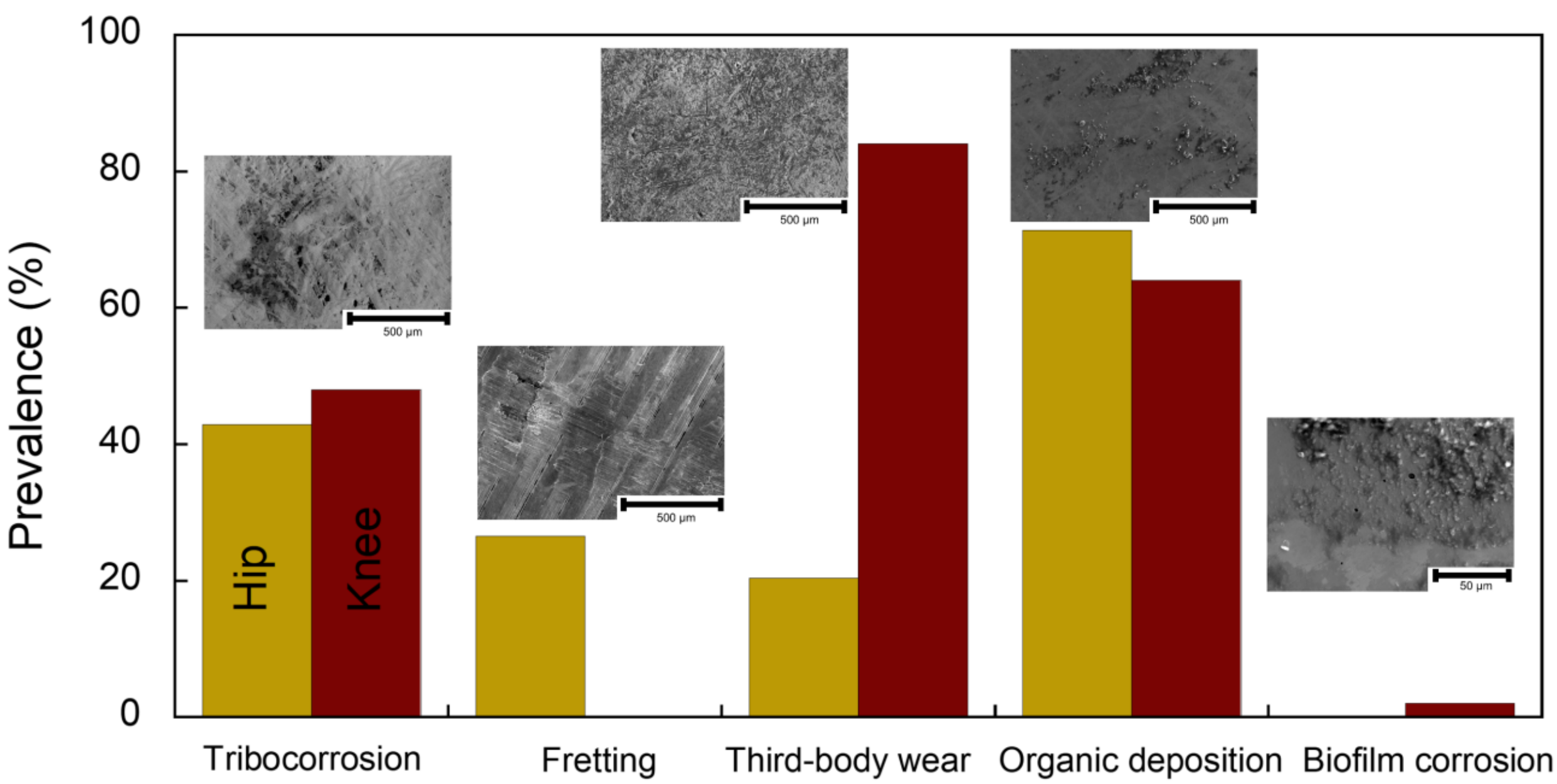

Fig. S8. The prevalence (%) of each damage type for 49 hip and 50 knee implants based on SEM analysis.

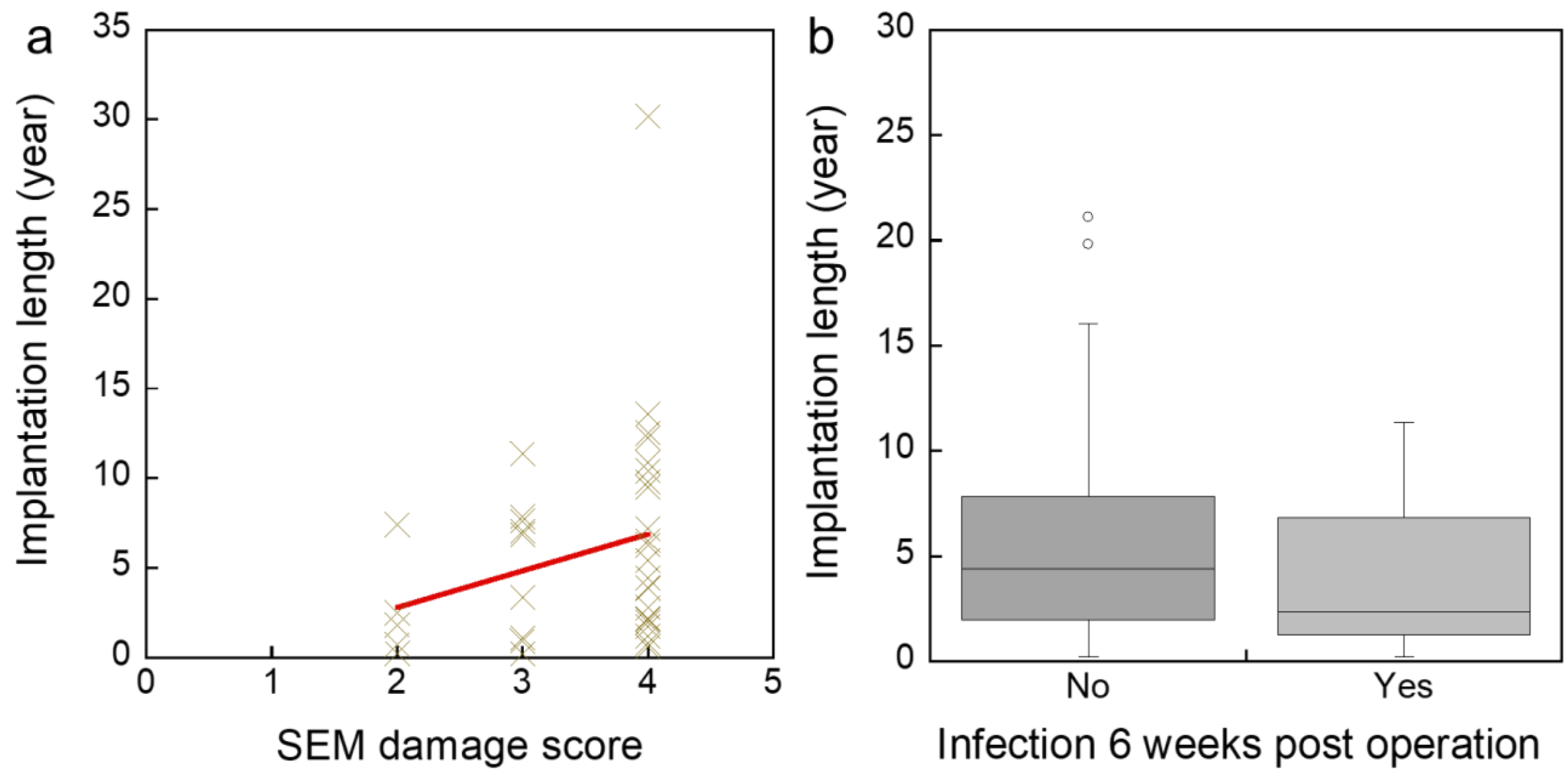

Fig. S9. a) Correlation between SEM and implantation length of knee implants (y = 1.27 + 2.04 x, R = 0.26, regression equation), b) Implantation length as a function of infection 6 weeks post operation for knee implants. Each box encloses 50% of the data with the median value of the variable displayed as a line. The top and bottom of the box mark the limits of ± 25% of the variable population. The lines extending from the top and bottom of each box mark the minimum and maximum values within the data set that fall within an acceptable range. The acceptable range is less than UQ + 1.5 * IQD or less than LQ – 1.5 * IQD, where UQ is the data value located halfway between the median and the largest data value, IQD stands for the distance between the Upper and Lower Quartiles (UQ – LQ), and LQ is the data value located halfway between the median and the smallest data value. Any value outside of this range, called an outlier, is displayed as an individual point.

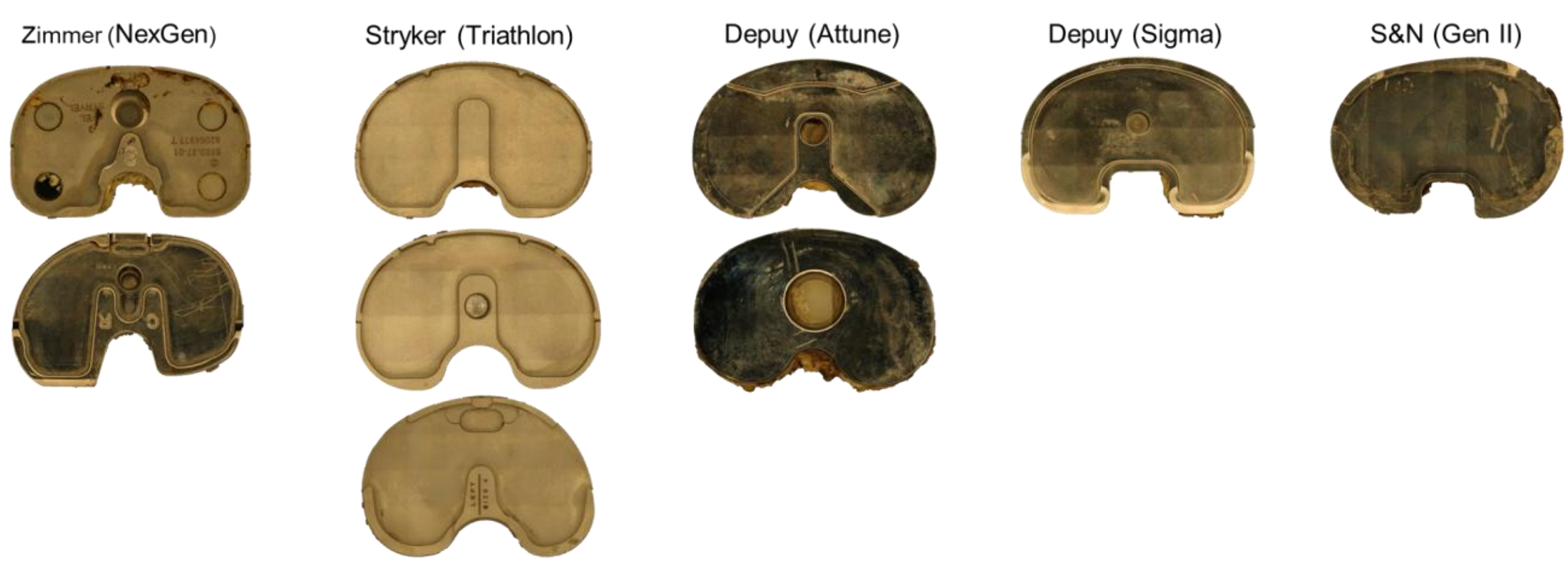

Fig. S10. Different kinds of implant systems from various manufacturers in the sample pool of 106 knee implants. S&N: Smith & Nephew, Gen II: Genesis II.

In this study, since each implant system represents a specific manufacturer, the Kruskal-Wallis test after analysis of 106 knee implants (n = 36 Zimmer, n = 21 DePuy, n = 45 Stryker, and n = 4 Smith & Nephew), revealed significant differences among manufacturers ($H = 49.63$, $df = 3$, $P < 0.001$) as well as systems ($H = 50.36$, $df = 4$, $P < 0.001$).

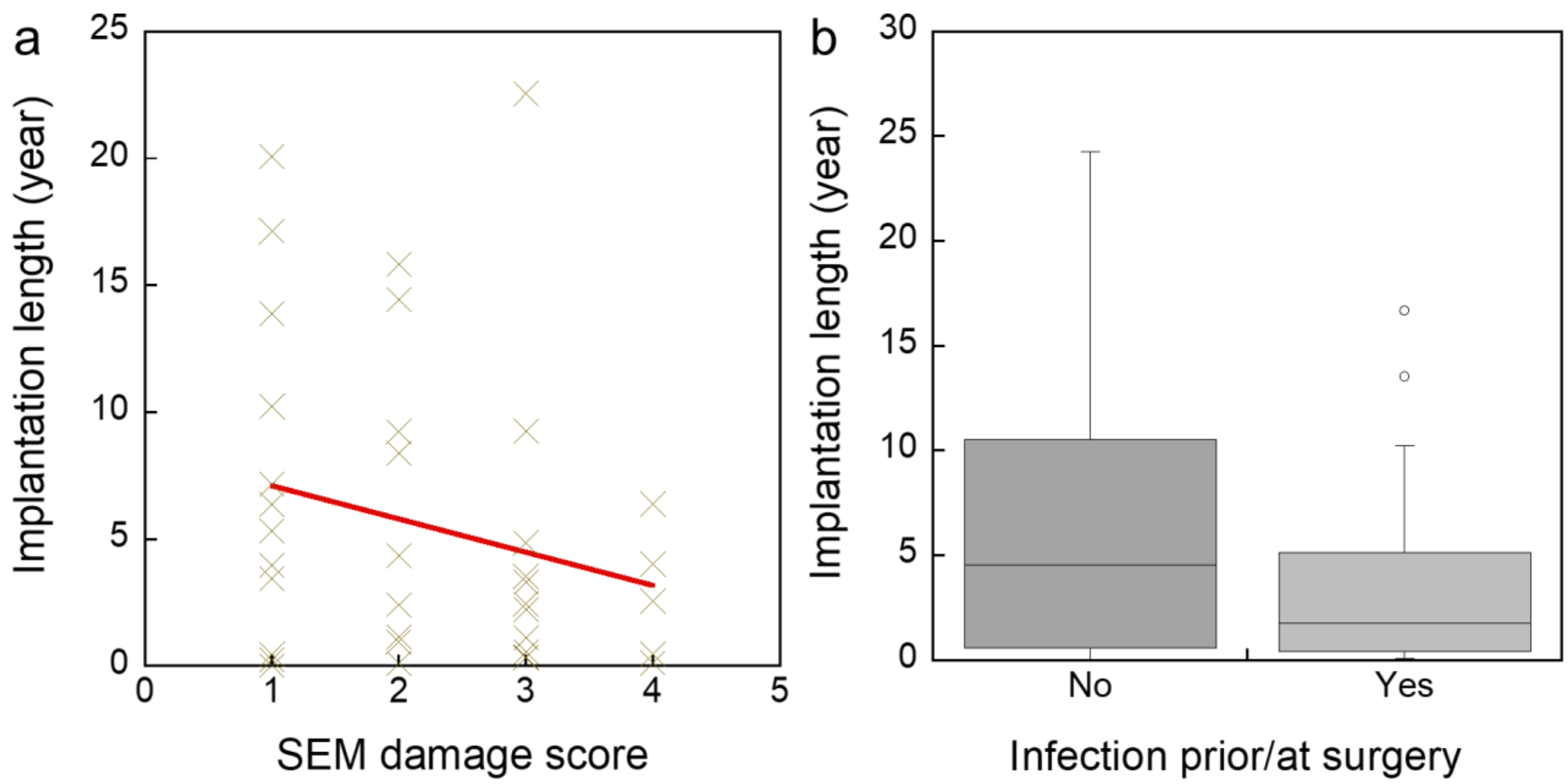

Fig. S11. a) Correlation between SEM and implantation length of hip implants (y = 8.48 – 1.30 x, R = 0.23, regression equation), b) Correlation between implantation length and infection prior to/at surgery for hip implants. Each box encloses 50% of the data with the median value of the variable displayed as a line. The top and bottom of the box mark the limits of ± 25% of the variable population. The lines extending from the top and bottom of each box mark the minimum and maximum values within the data set that fall within an acceptable range. The acceptable range is less than UQ + 1.5 * IQD or less than LQ – 1.5 * IQD, where UQ is the data value located halfway between the median and the largest data value, IQD stands for the distance between the Upper and Lower Quartiles (UQ – LQ), and LQ is the data value located halfway between the median and the smallest data value. Any value outside of this range, called an outlier, is displayed as an individual point.

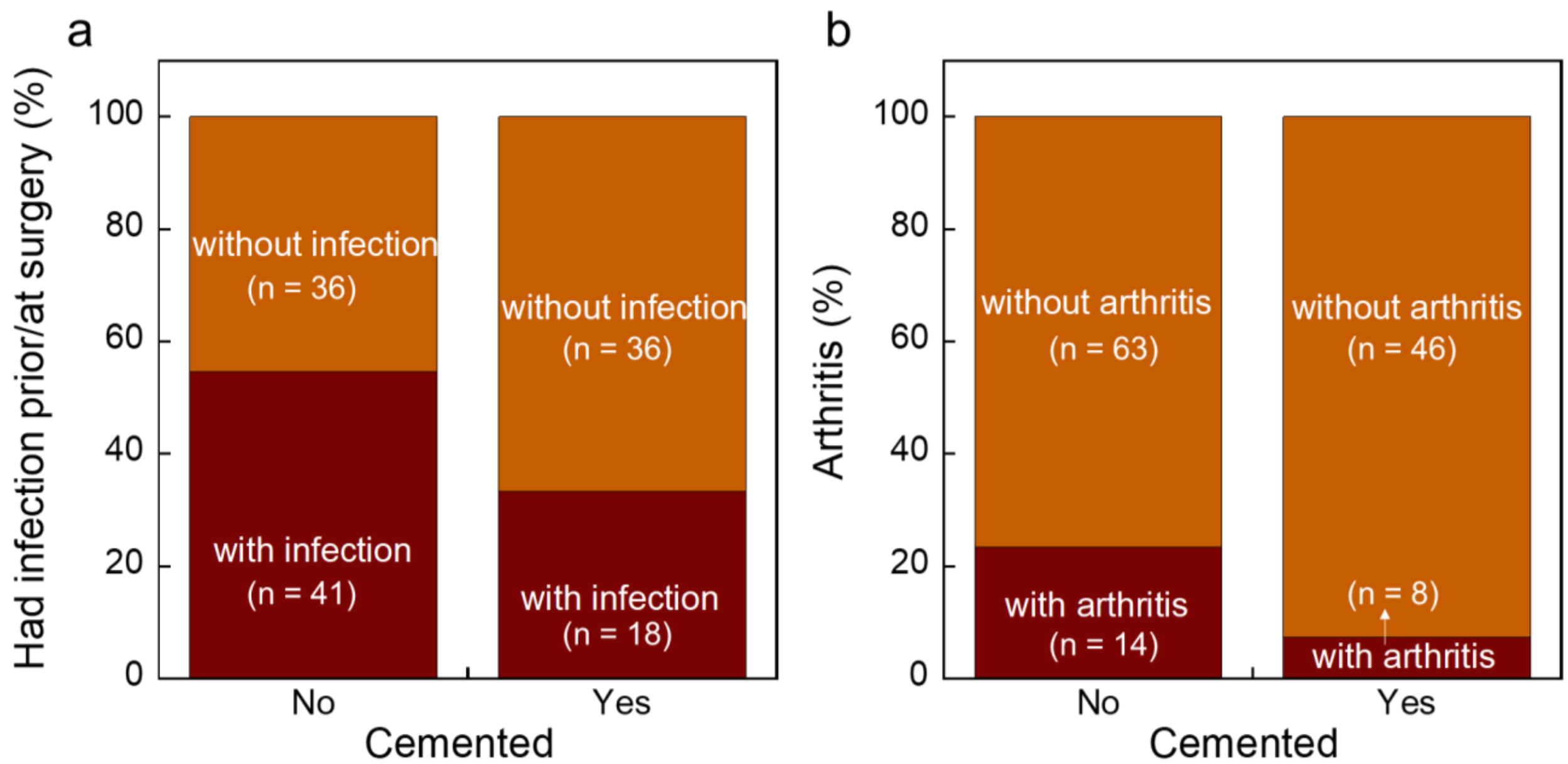

Fig. S12. a) Association between having an infection prior/at surgery and cementation status of hip implants, b) Association between having inflammatory arthritis and cementation status of hip implants. The number of each condition (n) is also reported.

Table S3. Correlation between hip implants and only knee implants with NexGen systems.

Ranks

Implant Type		N	Mean Rank	Sum of Ranks
SEM	Knee	18	53.33	960.00
	Hip	49	26.90	1318.00
	Total	67		
OM	Knee	36	114.82	4133.50
	Hip	135	78.31	10572.50
	Total	171		

Test Statistics[a]

	SEM	OM
Mann-Whitney U	93.000	1392.500
Wilcoxon W	1318.000	10572.500
Z	-5.117	-4.182
Asymp. Sig. (2-tailed)	0.000	0.000

a. Grouping Variable: Implant Type

Table S4. Correlation between hip implants and only knee implants with Attune systems.

Ranks

Implant Type		N	Mean Rank	Sum of Ranks
SEM	Knee	7	46.50	325.50
	Hip	49	25.93	1270.50
	Total	56		
OM	Knee	11	121.50	1336.50
	Hip	135	69.59	9394.50
	Total	146		

Test Statistics[a]

	SEM	OM
Mann-Whitney U	45.500	214.500
Wilcoxon W	1270.500	9394.500
Z	-3.235	-4.146
Asymp. Sig. (2-tailed)	0.001	0.000
Exact Sig. [2*(1-tailed Sig.)]	0.001[b]	

a. Grouping Variable: Implant Type,
b. Not corrected for ties.

Table S5. Correlation between hip implants and only knee implants with Triathlon systems.

Ranks

Implant Type		N	Mean Rank	Sum of Ranks
SEM	Knee	22	51.27	1128.00
	Hip	49	29.14	1428.00
	Total	71		
OM	Knee	45	72.80	3276.00
	Hip	135	96.40	13014.00
	Total	180		

Test Statistics[a]

	SEM	OM
Mann-Whitney U	203.000	2241.000
Wilcoxon W	1428.000	3276.000
Z	-4.316	-2.796
Asymp. Sig. (2-tailed)	0.000	0.005

a. Grouping Variable: Implant Type

Table S6. Correlation between hip implants and only knee implants with Sigma systems.

Ranks

Implant Type		N	Mean Rank	Sum of Ranks
SEM	Knee	2	38.00	76.00
	Hip	49	25.51	1250.00
	Total	51		
OM	Knee	10	107.15	1071.50
	Hip	135	70.47	9513.50
	Total	145		

Test Statistics[a]

	SEM	OM
Mann-Whitney U	25.000	333.500
Wilcoxon W	1250.000	9513.500
Z	-1.214	-2.822
Asymp. Sig. (2-tailed)	0.225	0.005
Exact Sig. [2*(1-tailed Sig.)]	0.285[b]	

a. Grouping Variable: Implant Type

b. Not corrected for ties.

Table S7. Correlation between hip implants and only knee implants with Genesis II systems.

Ranks

Implant Type		N	Mean Rank	Sum of Ranks
SEM	Knee	1	46.50	46.50
	Hip	49	25.07	1228.50
	Total	50		
OM	Knee	4	118.00	472.00
	Hip	135	68.58	9258.00
	Total	139		

Test Statistics[a]

	SEM	OM
Mann-Whitney U	3.500	78.000
Wilcoxon W	1228.500	9258.000
Z	-1.516	-2.562
Asymp. Sig. (2-tailed)	0.130	0.010
Exact Sig. [2*(1-tailed Sig.)]	0.160[b]	

a. Grouping Variable: Implant Type
b. Not corrected for ties.

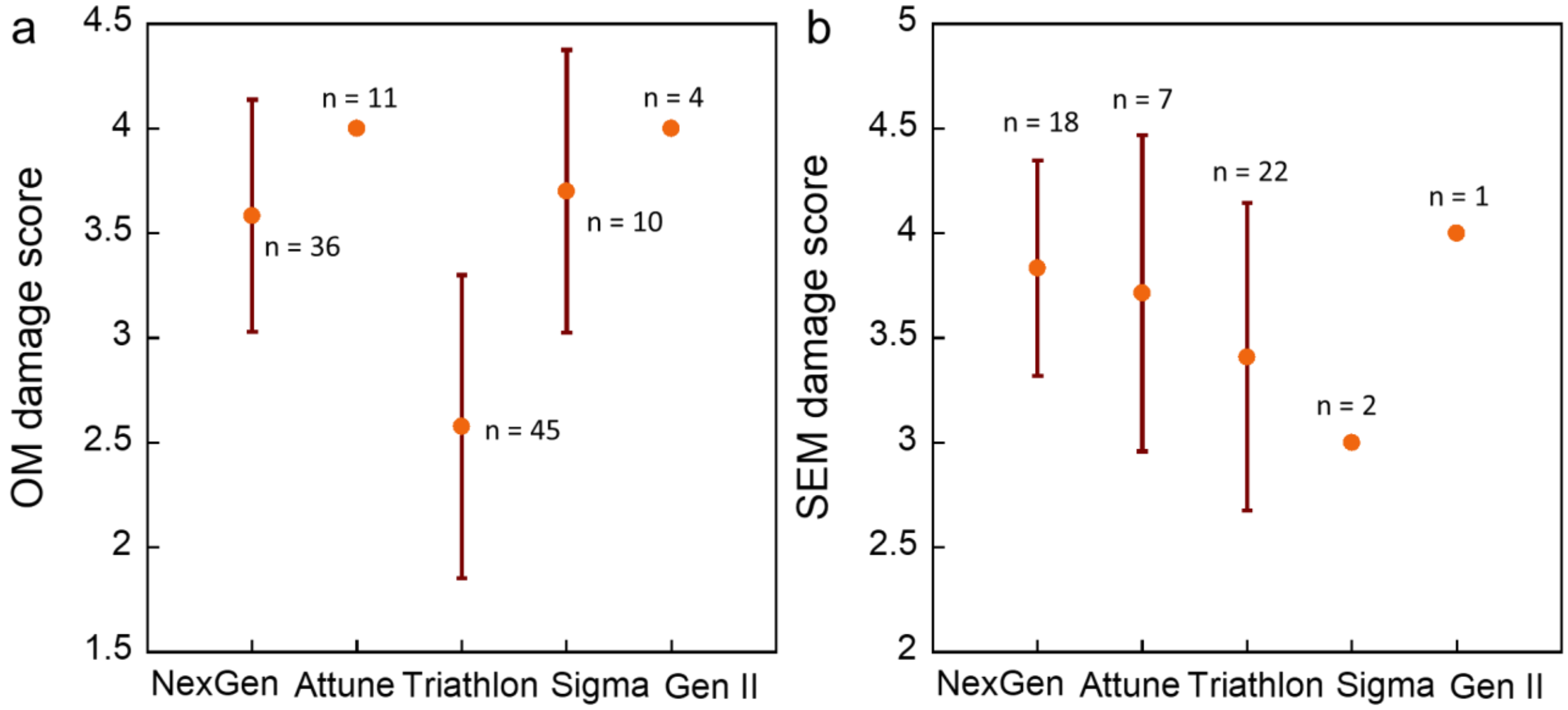

Fig. S13. a) Distribution of OM damage scores of knee implants for each implant system, b) Distribution of SEM damage scores of knee implants for each implant system. The number of each system (n) is also reported.

Tables S3-S7 and Figure S13 showed that SEM/OM damage scores remained significantly higher for knee implants when tibial baseplates with the NexGen and Attune systems were compared individually with those of hip implants. Triathlon systems still showed significantly higher SEM scores than hip implants, but their OM damage scores were significantly lower than hip implants. This implies that the optimized locking mechanism of the Triathlon system can, on a macroscopic scale, limit surface damage on the tibial part to a level even lower than that of the less-loaded hip implants. The SEM damage scores were not significantly different between hip and knee implants when only the Sigma system was considered, whereas OM damage scores were significantly higher in knee implants with this system than in hip samples. Finally, the comparison between knee implants with the Genesis II system and hip implants showed only a significant difference in OM damage scores, with knee implants having higher damage scores.

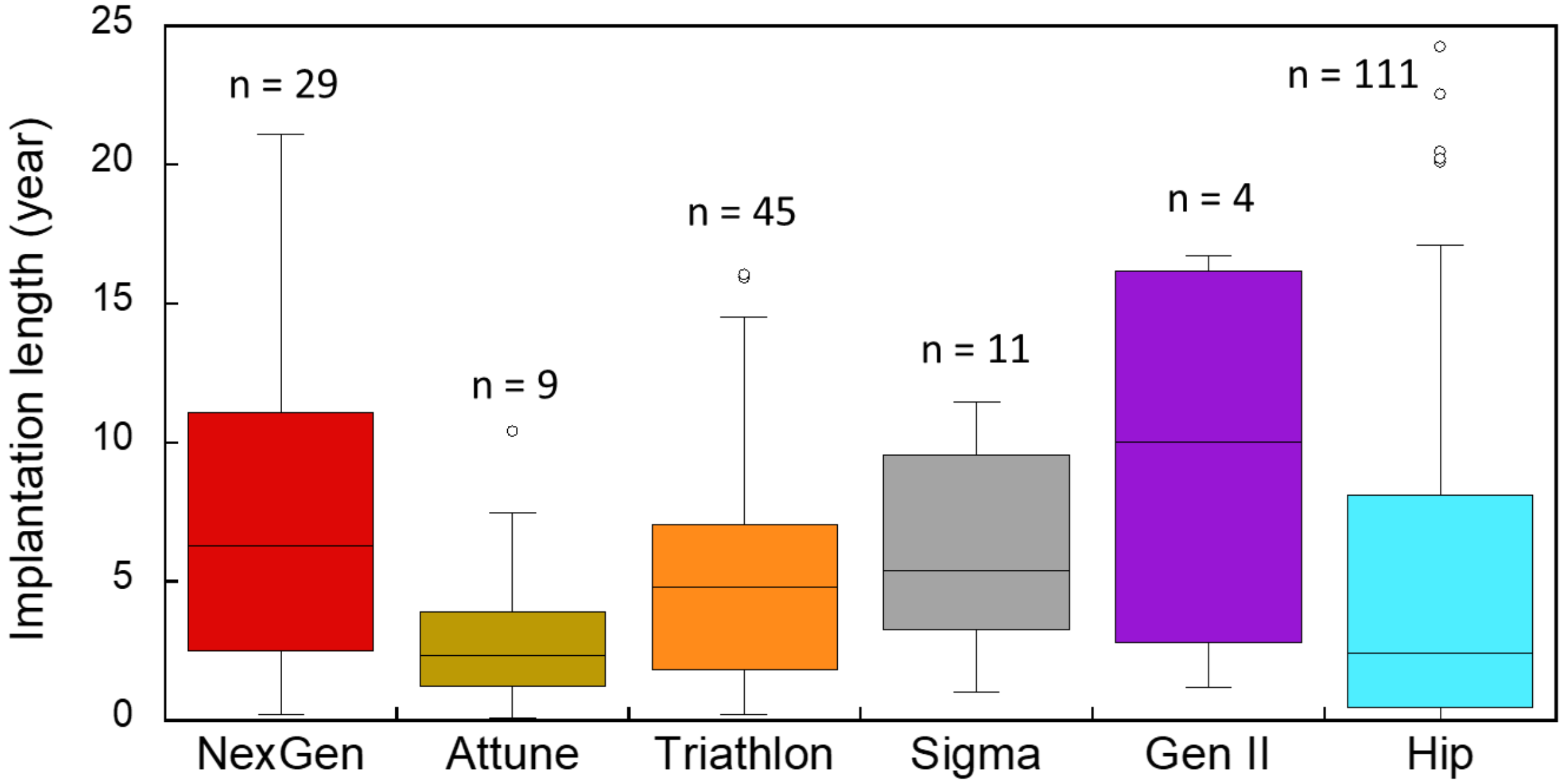

Fig. S14. Implantation length of each knee implants' systems and hip implants. The number of each knee implant system and hip implant (n) are reported. Each box encloses 50% of the data with the median value of the variable displayed as a line. The top and bottom of the box mark the limits of ± 25% of the variable population. The lines extending from the top and bottom of each box mark the minimum and maximum values within the data set that fall within an acceptable range. The acceptable range is less than UQ + 1.5 * IQD or less than LQ – 1.5 * IQD, where UQ is the data value located halfway between the median and the largest data value, IQD stands for the distance between the Upper and Lower Quartiles (UQ – LQ), and LQ is the data value located halfway between the median and the smallest data value. Any value outside of this range, called an outlier, is displayed as an individual point.
